# Dynamic modulation of intercellular adhesion mediated by matriptase-EPCAM/Trop-2 axes is critical for cell extrusion, division, and collective migration

**DOI:** 10.1080/19336918.2026.2663684

**Published:** 2026-05-05

**Authors:** Yu-Ling A. Kao, Qiaochu Wang, Dajun D. Lu, Shigeki Umemura, Vivien Lee, Ying Hsuan Tsai, Robert B. Barndt, Jehng‑Kang Wang, Michael D. Johnson, Chen-Yong Lin

**Affiliations:** a Lombardi Comprehensive Cancer Center, Department of Oncology, Medical Center, Georgetown University, Washington, DC, USA; b Department of Thoracic Oncology, National Cancer Center Hospital East, Bunkyo-ku, Japan; c School of Medicine, National Yang Ming Chiao Tung University, Yangming Campus, Taipei, Taiwan; d School of Medicine, National Defense Medical University, Taipei, Taiwan; e Department of Biochemistry, National Defense Medical University, Taipei, Taiwan

**Keywords:** Cell adhesion, cell division, cell extrusion, collective migration, EpCAM, matriptase, Trop-2

## Abstract

Epithelial dynamics require rapid remodeling of cell adhesion during extrusion, division, and migration. We identify a matriptase-dependent mechanism regulating the Ca^2+^- independent adhesion molecules EpCAM and Trop-2. Matriptase activation cleaves these CAMs into two-chain forms, promoting internalization and degradation, followed by replenishment through new synthesis. This turnover enables rapid adaptation of adhesion to environmental cues. Loss of matriptase disrupts this cycle, impairing epithelial integrity, extrusion, mitosis, and collective migration. Matriptase activity is tightly controlled by zymogen activation and inhibition by HAI-1, restricting CAM cleavage. Environmental factors, including pH, redox state, and chloride levels, modulate activation, linking external stimuli to adhesion remodeling. This protease-driven pathway provides a rapid, adaptable system for epithelial adhesion control.

## Introduction

Epithelial cell packing, the spatial arrangement of epithelial cells, results in continuous sheets of cells that line and protect both the internal and external surfaces of organs [1–4]. This organized structure is essential for the formation of functional epithelial tissues, serving as a protective barrier, regulating the permeability to and transport of molecules, and supporting various functions such as secretion, absorption, excretion, and sensation. Cell adhesion molecules (CAMs) facilitate the tight packing of epithelial cells, with E-cadherin and epithelial cell adhesion molecule (EpCAM) being two prominent examples [1,5–9]. Both are transmembrane glycoproteins that mediate cell-cell adhesion through homotypic interactions in either *trans* or *cis* configurations and connect indirectly to the cells’ actin filaments (F-actin) [10–12]. Despite their similarities, E-cadherin and EpCAM perform distinct functions. E-cadherin is a core component of adherens junctions (AJs) [13]; specialized adhesive structures observable under electron microscopy, whereas EpCAM is not associated with any defined junctional complexes [14]. The coexistence of junctional and non-junctional adhesions, which differ in their adhesive strength, supports the idea that EpCAM may enable cells to rapidly form and dissolve transient adhesions. These flexible contacts could then mature into more stable junctional adhesions mediated by E-cadherin. While both E-cadherin and EpCAM are capable of spontaneously initiating intercellular adhesion through homotypic interactions, the mechanisms that allow these adhesions to be rapidly weakened or dissolved remain poorly understood for both proteins. This lack of our understanding of a well-defined, adaptable mechanism for modulating adhesion dynamics presents a challenge in explaining how CAMs participate in essential epithelial processes such as cell division, extrusion, and collective migration. In these contexts, adhesions must be quickly and precisely modulated, either weakened or reinforced, to maintain epithelial integrity during events like cell death or division, and to ensure coordinated directional movement of groups of cells [15–17].

While E-cadherin can detect and respond to changes in cell surface mechanics caused by cell division or death [18–21], the limited N-terminal cleavage of EpCAM has the potential to play a pivotal role in facilitating the rapid and adaptable modulation of cell-cell adhesion [22]. Limited proteolytic cleavage is a common regulator approach in many systems and for example serves as a crucial regulatory mechanism for activating enzymes from their zymogen forms and growth factors from their precursor states [23–26]. This process also occurs naturally with EpCAM, though the functional consequences on EpCAM-mediated biology remain unclear. The key cleavage site has been identified at Arg-80, within an amino acid stretch in the Thyroglobulin type-1 domain which is stabilized by a disulfide bond [22]. As a result, this cleavage converts mature single-chain EpCAM into a disulfide-linked two-chain form. In spite of remaining on the cell surface, the impact of this cleavage on EpCAM’s properties, such as its homotypic interactions or connection to F-actin, remains largely uncharacterized. The formation of two-chain EpCAM can be induced by treating cells with various secreted proteases, each with different cleavage specificities, including trypsin, plasmin, thrombin, chymotrypsin, V8 protease, proteinase K, and papain [27]. Notably, however, this characteristic ‘limited’ proteolytic cleavage of EpCAM occurs only when it is present on the intact cell membrane, whereas immunopurified EpCAM in solution is completely hydrolyzed by these proteases. This suggests that the EpCAM molecule is generally well protected on the cell membrane, except for the stretch of amino acid between Cys-66 and Cys-99, which remains highly exposed and particularly susceptible to proteolytic cleavage. More recently, it has been shown that the serine protease domain of the type 2 transmembrane serine protease matriptase, unlike the glycosylphosphatidylinositol (GPI)-anchored serine protease prostasin, can facilitate limited, but not extensive, cleavage of recombinant EpCAM in solution [28,29]. This finding suggests a potential role for cellular serine proteases in the limited cleavage of EpCAM. However, this may instead reflect the intrinsic preference of trypsin-like serine proteases, such as the recombinant matriptase serine protease domain, to cleave substrates at arginine and/or lysine residues, which occur abundantly in most proteins, in solution [30]. Interestingly, despite their closely related substrate specificities and shared inhibitors, hepatocyte growth factor activator inhibitor-1 (HAI-1) and HAI-2, matriptase and prostasin display markedly different capacities to cleave EpCAM [31]. Further complicating this biochemical context, A Disintegrin And Metalloproteinase 10 (ADAM10) has been reported to cleave Trop-2, a close EpCAM homolog, that undergoes N-terminal processing at an arginine residue within the thyroglobulin type-1 domain [32]. However, this conclusion was based on studies using a Trop-2 double mutant (A87-A88) derived from the wild-type R87-T88 sequence, under the assumption that alanine substitution would prevent ADAM10-mediated cleavage at Arg-87. In fact, this mutation may instead enhance susceptibility to ADAM10, given the enzyme’s small S1 pocket and its known preference for alanine at the P1 position [33]. Consequently, ADAM10 is unlikely to mediate physiological N-terminal cleavage of Trop-2, and proposed roles for ADAM10-dependent Trop-2 cleavage in cancer growth and metastasis lack a valid experimental foundation.

In spite of its potent trypsin-like activity, which enables the extensive hydrolysis of extracellular matrix components [30,34], the proteolytic activity of matriptase is tightly regulated and the free active form of the enzyme has an exceptionally short half-life [35–37]. This tight control results from the precisely coordinated zymogen activation process, which generates enzymatically active matriptase, followed by the rapid formation of a stable enzyme-inhibitor complex with HAI-1, which effectively suppresses its proteolytic activity. This short half-life, likely in the second range, ensures that active matriptase can only perform limited proteolysis. Furthermore, the widespread expression of matriptase and EpCAM in epithelial cells, along with the localization of both molecules at the basolateral plasma membrane [38–41], suggests that matriptase is a candidate epithelial cell surface protease responsible for the generation of the two-chain form of EpCAM. Similarly, Trop-2, a structurally highly related protein, has also been reported to undergo cleavage by matriptase [29,42]. In this study, the biochemical enzyme-substrate relationship between matriptase and EpCAM (as well as Trop-2) is extensively explored. This functional characterization leads to the establishment of a matriptase-EpCAM axis in simple epithelia and a matriptase-Trop-2 axis in stratified squamous epithelia. These pathways constitute a tightly regulated cycle of adhesion remodeling, beginning with matriptase activation, followed by cleavage and internalization of EpCAM or Trop-2, and concluding with the reestablishment of adhesion through the synthesis of new CAMs. Functional studies reveal that loss of this cleavage-internalization mechanism, achieved by targeted deletion of matriptase, impairs critical epithelial processes such as cell extrusion, mitosis, and coordinated migration. In summary, the protease-CAM axis provides a versatile mechanism for the dynamic regulation of epithelial adhesion. These pathways likely act in concert with more stable junctional systems such as E-cadherin to balance flexibility and structural stability, ensuring proper epithelial function under both homeostatic and stress conditions.

## Materials and methods

### Reagents

Alexa Fluor® 594 goat anti-mouse IgG (Catalog # A-11012) and Alexa Fluor® 594 goat anti-rabbit IgG (Catalog # A-11012) were sourced from ThermoFisher Scientific (Waltham, MA). Alexa Fluor® 488 phalloidin (Catalog # 23115) was acquired from AAT Bioquest (Sunnyvale, CA). Crystal violet and 4,’6-diamidino-2-phenylindole (DAPI) were obtained from Sigma-Aldrich (St. Louis, MO). Thymidine was obtained from Research Product International (Mt. Prospect, IL). Protoblue Safe Coomassie G-250 stain was purchased from National Diagnostics (Atlanta, GA).

### Cell cultures

All cell lines and their corresponding knockout (KO) variants used in this study were cultured in Dulbecco’s Modified Eagle Medium (DMEM) supplemented with 10% fetal bovine serum (FBS), except for the IU-TAB1 human thymoma cells and RPMI8226 human multiple myeloma cells, which were maintained in RPMI1640 medium supplemented with 10% FBS. All cells were incubated at 37°C in a humidified atmosphere containing 5% CO_2_. The generation and characterization of the matriptase KO cell lines, in which the *ST14* gene was disrupted via CRISPR, have been previously described [43–45].

### Western blotting and antibodies

Cells were harvested by scraping on ice and lysed in PBS containing 1% Triton X-100 and 1 mM 5,5-dithio-bis-(2-nitrobenzoic acid) (DTNB). Following centrifugation to remove insoluble debris, protein concentrations in the supernatants were measured using the Bio-Rad Protein Assay Dye Reagent (Bio-Rad Laboratories, Hercules, CA, USA). Lysates were diluted in 5× SDS sample buffer without reducing agents. For reducing conditions, 1 µl of 2-mercaptoethanol was added to 25 µl of sample, followed by heating at 95°C for 5 minutes. Equal amounts of protein were separated by 10% SDS-PAGE and transferred onto 0.45 µm polyvinylidene fluoride (PVDF) membranes. Membranes were probed with the indicated primary monoclonal antibodies (mAbs), followed by incubation with HRP-conjugated anti-mouse IgG (Catalog # 074–1806) or anti-rabbit IgG (Catalog # 5220–0337) secondary antibodies (0.1 µg/ml, SeraCare, formerly Kirkegaard & Perry Laboratories). Detection was performed using Pierce™ ECL Western Blotting Substrate (Thermo Scientific, REF 32106) and visualized on x-ray film or using an Amersham Imager 600. Primary mAbs included M24 (2 µg/ml), M32 (2 µg/ml), and M69 (2 µg/ml) (targeting matriptase), and M19 (2 µg/ml) (targeting HAI-1), were developed and characterized as described in our previous publications [46–48]. The commercial antibodies used in this study were: mouse mAb 9C4 (5 µg/ml) against EpCAM (BioLegend, Cat# 3242002), used exclusively for immunofluorescence studies; mouse mAb C-10 (4 µg/ml) against EpCAM (Santa Cruz, sc-25308), used for immunoblotting; and rabbit mAb EPR20043 against Trop-2 (0.3 µg/ml) (Abcam, Cat# ab214488), used for both western blotting and immunofluorescence.

### Immunofluorescent staining

The subcellular localization of EpCAM and Trop-2 was assessed using indirect immunofluorescence. Cells were seeded onto 18 mm round glass coverslips placed in 12-well plates and cultured overnight. After experimental treatments, cells were washed with PBS, fixed with 10% buffered formalin (Fisher Scientific) for 10 minutes, and then permeabilized with 0.5% Triton X-100 in PBS for 5 minutes.

Cells were incubated for 1 hour at room temperature with either anti-EpCAM monoclonal antibody 9C4 (5 µg/ml) or anti-Trop-2 monoclonal antibody EPR20043 (1.3 µg/ml) diluted in PBS containing 3% bovine serum albumin (BSA). After extensive PBS washes, cells were incubated for 60 minutes at room temperature with Alexa Fluor® 594-conjugated goat anti-mouse IgG (4 µg/ml) or goat anti-rabbit IgG (4 µg/ml), along with Phalloidin-iFluor 488 conjugate (1:1,000 dilution), all in 3% BSA/PBS. The nuclei were counterstained with 1 μg/ml DAPI, added to the incubation buffer during the final 5 minutes.

### CCLE database and statistical analysis tools

The Cancer Cell Line Encyclopedia (CCLE) provides RNA-seq-based gene expression data for over 1,000 cancer cell lines. This dataset is publicly accessible through the UCSC Xena browser (xena.ucsc.edu/), where the raw expression data have been uniformly processed using a standardized pipeline to reduce variability across different sources. This enables more reliable comparisons of gene expression across cell lines from various tissue origins. All gene expression values and ratios reported in this study were obtained from the CCLE dataset using the UCSC Xena platform and calculated based on RPKM values.

### Double thymidine block for cell synchronization, division, and extrusion

HaCaT human keratinocytes were synchronized using a double thymidine block. Cells were first treated with 16 mM thymidine in DMEM containing 10% FBS for 16 hours, then released into regular culture medium for 8 hours, followed by a second 16-hour thymidine treatment. To monitor progression post-synchronization, cells were released into fresh medium with 10% FBS and harvested at various time points (0, 0.5, 1, 2, 3, 4, 6, 8, and 24 hours). At each time point, cells were washed twice with PBS and lysed for subsequent analysis. Conditioned media were collected after the first block, the second block, and 24-hr after the release, and centrifuged at 400 × g for 10 minutes to isolate extruded cells. Cell debris was washed twice with PBS and lysed in RIPA buffer.

### Live-cell imaging of cell division and collective migration

To monitor cell division and collective migration, both parental and matriptase knockout (KO) HaCaT cells were subjected to live-cell imaging at 10-minute intervals for 16 hours and at 2 hours intervals for 24 hours, respectively, using a Nikon CSU-W1 SoRa confocal microscope. The cell division process was divided into two stages: Stage I, from abrupt morphological changes associated with cell rounding and ending around metaphase; and Stage II, from anaphase to the reintegration of daughter cells with neighboring cells. The averaged time to complete each stage was calculated from the analysis of 60 dividing cells. For statistical comparison, unpaired t-test was performed using GraphPad Prism 10. Data are presented as the mean ± SEM. For the scratch wound healing assay, the cells were grown to near confluency before creating a wound in the monolayer using a 1,000 µL pipette tip. Approximately three hours after wounding, live-cell imaging was initiated at 2-hour intervals over a 24-hour period. To quantify migration rates, the wound area was measured using ImageJ. The change in wound area (Y-axis) from time 0 to each subsequent time point (X-axis) was plotted, and linear regression analysis was performed using GraphPad Prism 10. The slope of the resulting trend line was used to represent the rate of collective migration.

## Results

### The N-terminal cleavage of EpCAM is induced concurrently with the induction of matriptase zymogen activation

**Figure 1.**
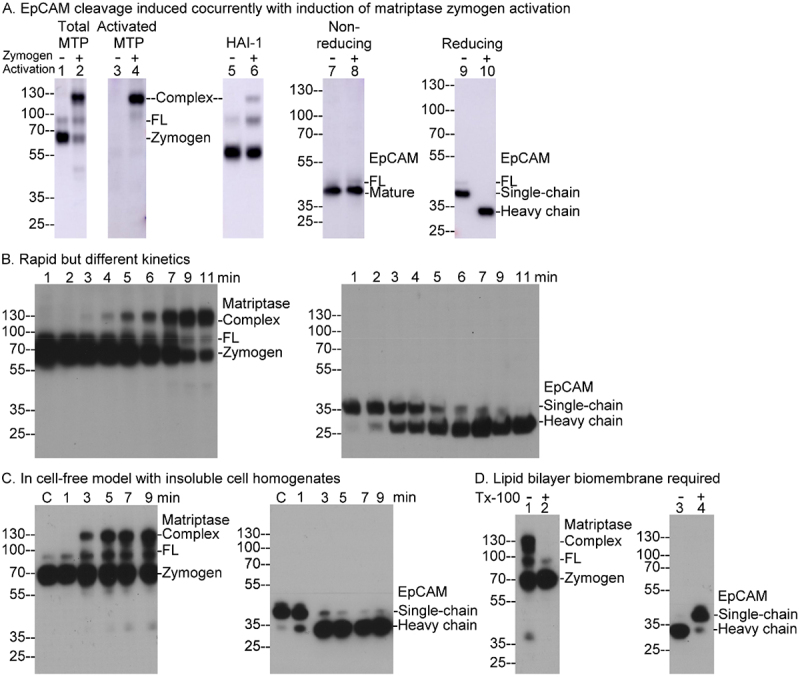
Concurrent induction of matriptase zymogen activation and EpCAM cleavage. A. T-47D breast cancer cells were treated with either a pH 6 phosphate buffer to induce matriptase zymogen activation for 15 minutes (Zymogen Activation, +) or PBS as a non-activation control (Zymogen Activation, -). Equal amounts of cell lysate proteins were analyzed via immunoblotting for total matriptase using the mAb M32 (lanes 1 and 2), activated matriptase using the mAb M69 (lanes 3 and 4), HAI-1 using the mAb M19 (lanes 5 and 6), and EpCAM using an EpCAM-specific mAb (lanes 7–10). Immunoblots were performed under non-reducing/non-boiled conditions, except for EpCAM in lanes 9 and 10, which were analyzed under reducing/boiled conditions.B. And C. HaCaT human keratinocytes (b) and the insoluble fractions from homogenized T-47D breast cancer cells (C) were exposed to a pH 6 phosphate buffer for the indicated times. Equal amounts of proteins were analyzed by immunoblotting for matriptase under non-reducing/non-boiled conditions (left panels) and for EpCAM under reducing/boiled conditions (right panels).D. Insoluble fractions from homogenized T-47D breast cancer cells were treated with pH 6 buffer either in the absence (lanes 1 and 3) or presence (lanes 2 and 4) of 1% Triton X-100 for 15 minutes. Equal amounts of proteins were analyzed by immunoblotting for matriptase under non-reducing/non-boiled conditions (lanes 1 and 2) and for EpCAM under reducing/boiled conditions (lanes 3 and 4). The various forms of matriptase, HAI-1, and EpCAM are indicated. MTP represents matriptase, and FL denotes full-length.

The N-terminal cleavage within the Thyroglobulin Type 1 domain of EpCAM involves proteolysis that must be tightly regulated and limited, resulting in the formation of two fragments that remain on the cell surface, held together by a disulfide bond. Matriptase is a likely candidate for the protease responsible for the cleavage due to its colocalization with EpCAM on the surface of epithelial cells and its tightly regulated tryptic activity. Matriptase is synthesized as a zymogen and only gains its enzymatic activity after undergoing zymogen activation to become an active enzyme. We have previously reported that brief exposure of cells to a pH 6 buffer efficiently and rapidly triggers matriptase zymogen activation [49–52]. Once activated, the newly generated enzymatically active matriptase is quickly inhibited by HAI-1 through the formation of a stable enzyme-inhibitor complex. As demonstrated in Figure 1(A), the 70-kDa matriptase zymogen was rapidly converted into a 120-kDa activated matriptase-HAI-1 complex following a 15-minute exposure of the cells to a pH 6.0 buffer (Figure 1(A), Total MTP, lane 2 versus lane 1). Notably, this enzyme-inhibitor complex, but not zymogen matriptase, was detected by the activated matriptase-specific mAb M69 (Figure 1(A), Activated MTP, lanes 3 and 4). Additionally, the enzyme-inhibitor complex and HAI-1 monomer were specifically detected by the HAI-1 mAb M19 (Figure 1(A), HAI-1, lanes 5 and 6). The presence of the matriptase-HAI-1 complex, rather than 70-kDa free, active matriptase, further supports the notion that enzymatically active matriptase has a very short lifespan. These findings demonstrate that transient acid exposure effectively induces robust matriptase zymogen activation and highlight the extremely short half-life of active matriptase due to rapid HAI-1 mediated inhibition. It is important to note that the enzyme-inhibitor complex remains stable in 1% SDS, and that SDS-PAGE was conducted under non-reducing/non-boiled conditions.

To determine whether matriptase plays a role in EpCAM’s N-terminal cleavage, the protein band profile for EpCAM before and after the induction of matriptase zymogen activation was analyzed using immunoblotting with an anti-EpCAM antibody after SDS-PAGE under either non-reducing or reducing conditions. Prior to transient acid exposure, EpCAM was detected as a band of approximately 38-kD, consistent with the single-chain form of the protein, with the apparent size of the band being the same under either non-reducing or reducing conditions (Figure 1(A), lanes 7 and 9). In contrast, following the induction of matriptase zymogen activation, EpCAM was detected as what appears to be a disulfide-linked two-chain form. This conclusion is based on its appearance as a 38-kDa band under non-reducing conditions (Figure 1(A), lane 8) and as a smaller 32-kDa fragment under reducing conditions (Figure 1(A), lane 10). The 32-kDa band corresponds to the heavy chain (the larger fragment) of two-chain EpCAM, containing the epitope recognized by the EpCAM mAb. The presence of two-chain EpCAM indicates that a limited proteolytic cleavage within the thyroglobulin type 1 domain of EpCAM can occur concurrent with the induction of matriptase zymogen activation.

In the past, investigators have used different conventions to refer to the various forms of EpCAM, and so for clarity it is important to explain the nomenclature regarding this protein. EpCAM is synthesized as a full-length (FL) protein that undergoes co-translational and post-translational modifications, including the removal of signal peptides resulting in the generation of its 38-kDa ‘mature and functional’ form, which mediates homotypic interactions. Mature EpCAM undergoes additional N-terminal cleavage within the Thyroglobulin type 1 domain to form a disulfide-linked two-chain structure. Thus, the 38-kDa band represents the mature form, which may exist as either a single-chain or two-chain variant. Some investigators have referred to the single-chain form of this 38-kDa species as full-length EpCAM, which is confusing [28,29]. In this study, what is more conventionally referred to as full-length EpCAM, the precursor form, was observed as a minor species slightly larger than the mature form (Figure 1(A), labeled as FL). Thus, the 38-kDa single-chain EpCAM observed here is not the full-length form of the protein. A similar nomenclature confusion was also for Trop-2 [29,42].

### EpCAM doesn’t contribute to matriptase autoactivation and must be anchored to the lipid bilayer biomembrane for the rapid induction of N-terminal cleavage

Matriptase zymogen activation involves cleavage at Arg-614 within the activation motif [30,53,54], which is mediated by the intrinsic activity of the matriptase zymogen itself, representing an unusual mechanism for zymogen activation known as autoactivation [55]. Matriptase autoactivation is believed to occur through a zymogen-activating-zymogen mechanism, potentially facilitated by interactions among matriptase zymogen molecules and possibly other associated proteins. If EpCAM plays a role in matriptase autoactivation and serves as more than a downstream substrate, one would expect the kinetics of EpCAM cleavage to parallel those of matriptase zymogen activation. If on the other hand, EpCAM is simply a substrate, one might expect more rapid kinetics since a single active protease molecule can cleave multiple substrate molecules. To assess this question, we evaluated the time course of these processes. The 120-kDa matriptase-HAI-1 complex was detectable as early as 3 minutes after acid exposure and continued to form at a relatively steady rate over the time course (Figure 1(B), left panel). As the level of the 120-kDa matriptase-HAI-1 complex increased, the level of the 70-kDa matriptase zymogen correspondingly decreased. In contrast, rapid EpCAM cleavage occurred between 3 and 5 minutes after acid exposure, with the majority of single-chain EpCAM being converted to its two-chain form within that timeframe (Figure 1(B)). By 2 minutes post acid exposure, only a small amount of two-chain EpCAM had been generated, likely produced by the initial traces of active matriptase, which remained below detectable levels (Figure 1(B), lanes for 2 min). It appears that once matriptase zymogen activation is triggered, the nascent active matriptase rapidly and efficiently cleaved single-chain EpCAM before being inhibited by HAI-1. Consequently, the two-chain EpCAM accumulated at a much faster rate than the activated matriptase. Since matriptase zymogen activation continued to occur 7 minutes after acid exposure, even when the single-chain EpCAM was nearly fully cleaved, this suggests that single-chain EpCAM is not essential for matriptase autoactivation. Single-chain EpCAM, therefore, appears to be simply a downstream substrate rather than a factor involved in, or essential for, matriptase zymogen activation.

Anchorage to a lipid bilayer bio-membrane has previously been shown to be essential for acid-induced matriptase zymogen activation [51]. The bio-membrane is believed to act as a platform facilitating the protein-protein interactions required for matriptase autoactivation. Membrane anchorage was also found to be essential for the induced cleavage of EpCAM (Figure 1(C)). Using insoluble fractions prepared from cell homogenates, both matriptase zymogen activation and EpCAM cleavage occurred with faster kinetics after acid exposure compared to live cells (Figure 1(C)). However, while matriptase zymogen activation was less efficient in terms of the percentage of zymogen activation, EpCAM cleavage occurred with similar efficiency as observed in the live, whole-cell model. When the nonionic detergent Triton X-100 was added to the pH 6.0 buffer at a dilution of 1%, neither matriptase activation nor EpCAM cleavage was induced (Figure 1(D), lane 1 versus lane 2 and lane 3 versus lane 4). This suggests that bio-membrane anchorage is essential for both matriptase zymogen activation and EpCAM cleavage.

### Matriptase is a long-sought primary protease responsible for generating two-chain EpCAM

**Figure 2.**
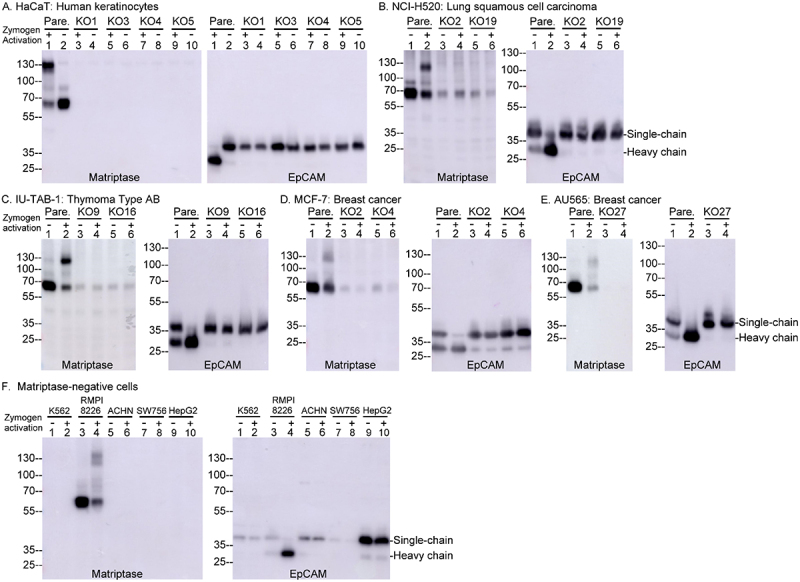
Matriptase is essential for acid-induced EpCAM cleavage and contributes to its endogenous processing. Five human epithelial cell lines (panels A–E, pare = parental) and their corresponding matriptase-deficient variants (panels A-E, KO = knockout), as indicated, as well as four matriptase-negative cell lines and RPMI 8226 human multiple myeloma cells (F), were transiently exposed to pH 6.0 buffer to induce matriptase zymogen activation and EpCAM cleavage (Zymogen Activation, +) or treated with PBS as non-activation controls (Zymogen Activation, -). Equal amounts of cell lysate proteins from each condition were subjected to immunoblot analysis for total matriptase using the mAb M32 under non-reducing/non-boiled conditions (left panels) and for EpCAM using the EpCAM mAb under reducing/boiled conditions (right panels). The single-chain and heavy chain of EpCAM are indicated.

The functional relationship between matriptase and EpCAM was further examined to determine whether matriptase is essential for EpCAM cleavage. This analysis included six matriptase-positive cell lines, from which 11 matriptase-deficient variants had been generated using CRISPR technology, as well as four naturally matriptase-negative cell lines (Figure 2). The six matriptase-positive cells, including human keratinocytes (Figure 2(A), lanes 1), human lung squamous cell carcinoma cells (Figure 2(B), lanes 2), human thymoma cells (Figure 2(C), lanes 2), human breast cancer cells (Figures 2(D,E), lanes 2), and human multiple myeloma cells (Figure 2(F), lanes 4), all responded to transient acid exposure by activating matriptase and producing two-chain EpCAM. In contrast, neither the 11 matriptase-deficient variants nor the four matriptase-negative cell lines exhibited increased levels of two-chain EpCAM following acid exposure. This finding suggests that matriptase plays a necessary role in acid-induced EpCAM cleavage.

Two-chain EpCAM has been reported in the literature to be present in cultured cells at varying levels in different cell types and even under different culture conditions. This natural variation in baseline EpCAM cleavage was also observed among the six matriptase-positive cell lines examined (Figure 2, EpCAM, Zymogen activation -), with the levels of two-chain EpCAM ranged from being barely detectable to comprising roughly half of the total EpCAM expressed. In contrast, the baseline level of two-chain EpCAM was negligible in all of the 11 matriptase-deficient variants (Figure 2, EpCAM, Zymogen activation -), underscoring the critical role of matriptase in generating endogenous two-chain EpCAM. Trace levels of two-chain EpCAM were detected in some matriptase-deficient variants. These low levels might be explained by residual matriptase activity. However, the presence of very low levels of two-chain EpCAM in four inherently matriptase-negative cell lines, particularly HepG2 human hepatoma cells (Figure 2(F), EpCAM, lanes 9 and 10), suggests that EpCAM cleavage can also be mediated by other cellular proteases, as reported in the literature. Given that treatment with proteases of various cleavage specificities can generate two-chain EpCAM via cleavage within the Thyroglobulin type 1 domain [27], the detection of two-chain EpCAM in matriptase-negative cells is not unexpected. The trace amounts observed may simply reflect the high accessibility of the Thyroglobulin type 1 domain to proteolytic activity.

It is important to note that due to the rapid shedding of the matriptase-HAI-1 complex from the cell surface, the amount of complex detected in cell lysates represents only a fraction of the total activated matriptase, typically a small proportion in cells undergoing active matriptase zymogen activation [49,56,57]. Consequently, the endogenous level of two-chain EpCAM may not directly correlate with the levels of matriptase-HAI-1 complex detected in cell lysates. In summary, the essential role of matriptase in acid-induced EpCAM cleavage, along with its contribution to the presence of endogenous two-chain EpCAM, identifies matriptase as the long-sought key cellular protease responsible for converting single-chain EpCAM into its two-chain form.

### Matriptase is also the long-sought key protease responsible for the generation of two-chain Trop-2

**Figure 3.**
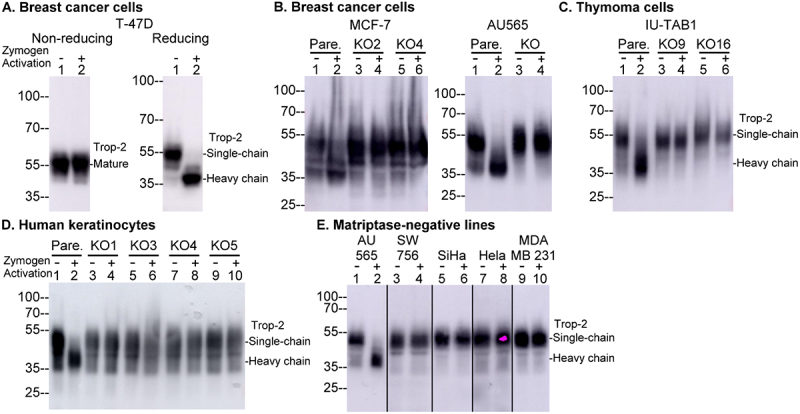
Matriptase is responsible for the acid-induced and endogenous generation of two-chain Trop-2 A. T-47D breast cancer cells were transiently exposed to a pH 6 phosphate buffer to induce matriptase zymogen activation (Zymogen Activation +, lanes 2) or PBS as the non-activation control (Zymogen Activation, -, lanes 1). Equal amounts of protein from cell lysates were analyzed by immunoblot for Trop-2 under either non-reducing/non-boiled conditions (left panel) or reducing/boiled conditions (right panel). B-F. Four different human epithelial cells (B.-D., pare.) with their matriptase deficient variants (KOs), as indicated, and four matriptase-negative cells plus one matriptase-positive control, as indicated (E.) were transiently exposed to a pH 6.0 buffer to induce matriptase zymogen activation (Zymogen Activation, +) or PBS as non-activation controls (Zymogen Activation, -). Equal amounts of cell lysate protein prepared from each group were analyzed by immunoblot for Trop-2.

Given the similarities in protein structure and expression pattern for Trop-2 with EpCAM, it seemed likely that this protein might also be a matriptase substrate and so we next set out to examine this possibility. In T-47D breast cancer cells, prior to matriptase zymogen activation, Trop-2 was primarily detected in its single-chain form, observed as a band of approximately 55-kDa under both non-reducing and reducing conditions (Figure 3(A), lanes 1). The conversion of single-chain Trop-2 to its two-chain form following robust matriptase zymogen activation was confirmed by the release and detection of a band consistent with the Trop-2 heavy chain observed exclusively under reducing conditions as a band of approximately 40-kDa (Figure 3(A), lanes 2). The essential role of matriptase in generating two-chain Trop-2 was further demonstrated by the absence of both endogenous baseline or acid-induced two-chain Trop-2 in nine matriptase-deficient variants derived from four different cell lines (Figure 3(B–D)). This lack of the presence of two-chain Trop-2 was also observed in four naturally occurring matriptase-negative cell lines, including three cervical cancer cell lines and one breast cancer cell line (Figure 3(E)).

### Extensive co-expression of matriptase with EpCAM and Trop-2 in various cancer cell lines

**Figure 4.**
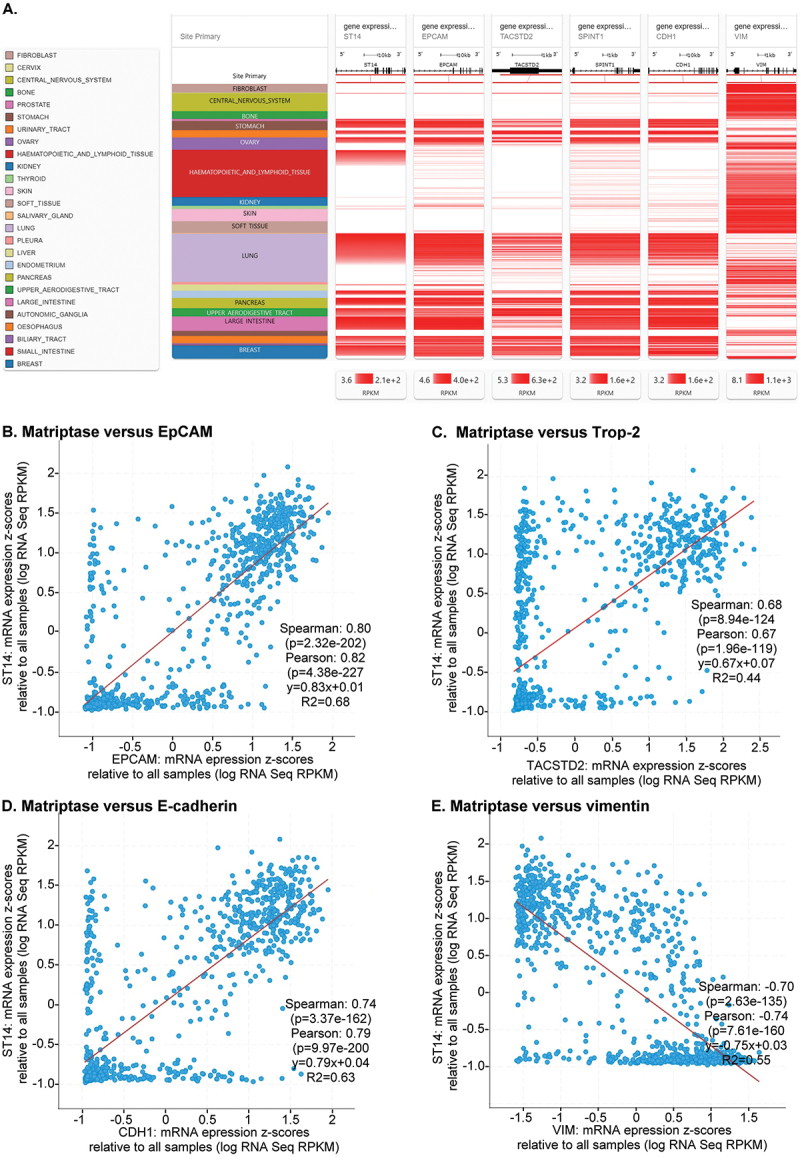
Frequent co-expression of matriptase, EpCAM, Trop-2, and HAI-1 in epithelial cells. A. The relative mRNA expression levels of matriptase (*ST14*), EpCAM (*EPCAM*), Trop-2 (*TACSTD2*), HAI-1 (*SPINT1*), E-cadherin (*CDH1*), and vimentin (*VIM*) were analyzed using data from the cancer cell Line Encyclopedia (CCLE) via the UCSC xena platform. Matriptase mRNA expression levels were ranked from high to low, and corresponding mRNA expression levels of EpCAM, Trop-2, HAI-1, E-cadherin, and vimentin were aligned accordingly. Each block represents 50 cell lines, and RNA-seq read count intensity is proportional to the shade of red in the visualization. B–E. Correlation analysis of matriptase mRNA expression (log RPKM) with EpCAM (b), Trop-2 (C), E-cadherin (D), and vimentin (E) mRNA expression across all cancer cell lines in the CCLE database. The calculated expression correlation coefficients are indicated on each plot.

For there to be significant broad pathophysiological relevance for these enzyme-substrate relationships, it would seem to require the widespread co-expression of EpCAM/Trop-2 with matriptase and HAI-1, the latter being critical for regulating EpCAM cleavage. To evaluate this question, the extent of the co-expression of the mRNAs for matriptase (*ST14*), EpCAM (*EPCAM*), Trop-2 (*TACSTD2*), and HAI-1 (*SPINT1*) in human cancer cell lines were analyzed using data from the Cancer Cell Line Encyclopedia (CCLE) database (https://sites.broadinstitute.org/ccle/). The CCLE database includes over a thousand human cell lines, representing the genomic diversity of human cancers. The epithelial marker E-cadherin (*CDH1*) and the mesenchymal marker vimentin (*VIM*) were also included in the analysis. The relative expression levels of these genes were compared and ranked by matriptase expression, grouped by organ or tissue of origin. As shown in Figure 4(A), high matriptase expression was observed in many cell lines derived from epithelia-rich organs and tissues. These cell lines also typically exhibited high levels of EpCAM, Trop-2, HAI-1, and E-cadherin. In contrast, cell lines with low or negligible matriptase expression typically exhibited similarly low levels of EpCAM, Trop-2, HAI-1, and E-cadherin, but expressed significant levels of vimentin. This expression profile suggests that matriptase, HAI-1, EpCAM, and Trop-2, like E-cadherin, are reliable markers for cells of predominantly epithelial origin. An intriguing exception to this observation is the apparently ectopic expression of matriptase in certain hematological cancer cell lines, where the correlation with HAI-1 and EpCAM expression was less consistent, and expression of Trop-2 and E-cadherin is essentially absent. Interestingly, these hematological cancer cell lines often express HAI-2, another potent biochemical inhibitor of matriptase. As we have previously reported, however, due to its predominant intracellular localization, HAI-2 is a less effective regulator of matriptase activity at the cellular level [58].

These expression patterns were further analyzed quantitatively by calculating the correlation coefficients between matriptase and the four other proteins. Across more than 1,000 cell lines, the data showed that cells with high matriptase expression frequently also exhibited high levels of EpCAM (Figure 4(B)), Trop-2 (Figure 4(C)), and E-cadherin (Figure 4(D)), and vice versa. The expression correlation coefficients for EpCAM and E-cadherin, determined using both Spearman and Pearson correlation tests, were approximately 0.75–0.8, indicating strong co-expression with matriptase in most cells (Figure 4(B,D)). Although the correlation coefficient for Trop-2 was lower (less than 0.7), the co-expression with matriptase was still considered moderately high (Figure 4(C)). In contrast, the strong negative correlation coefficient between matriptase and the mesenchymal marker vimentin reflects their almost mutually exclusive expression patterns (Figure 4(E)). These correlations are consistent with a functional link between matriptase, EpCAM, and Trop-2, and as key components of epithelial cells. Furthermore, partial co-expression can also be observed in some hematological cancer cells.

### Differences in the expression of the members of the matriptase-EpCAM/Trop-2 axes are associated with variations in epithelial morphology and function

**Figure 5.**
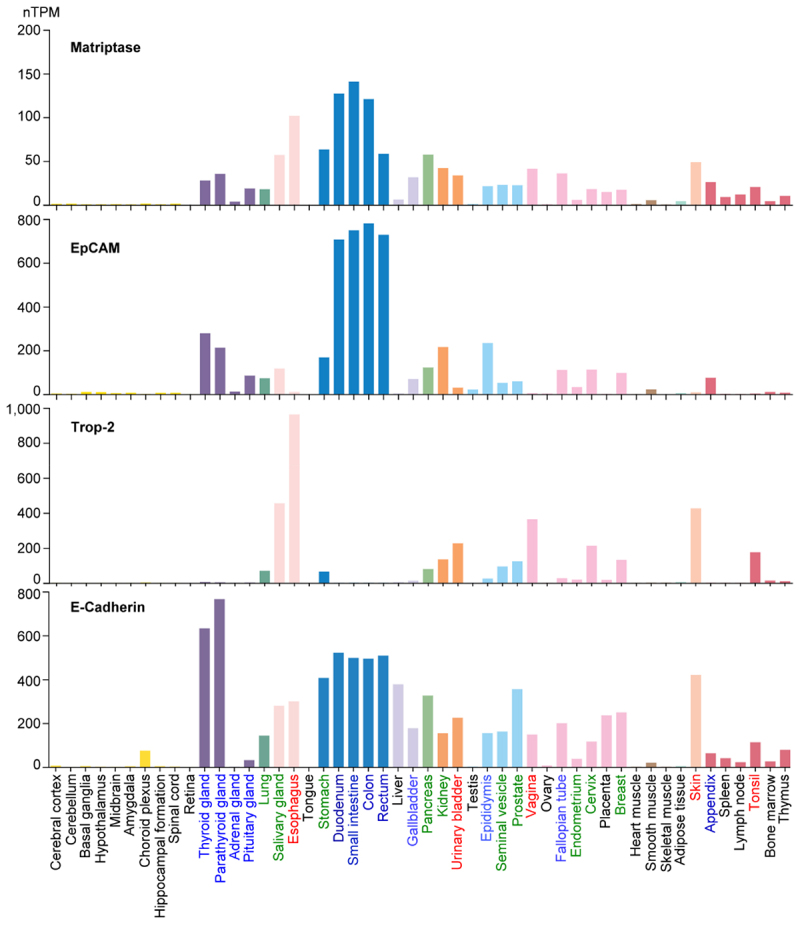
Differential predominance of the matriptase-EpCAM and matriptase-Trop-2 axes across different epithelial morphologies and functions. The relative mRNA expression levels of matriptase, EpCAM, Trop-2, and E-cadherin in various human tissues, as indicated, were compared using data from the human protein Atlas. Based on the patterns of EpCAM and Trop-2 expression, the tissues were categorized into four groups, represented by different colors: blue for EpCAM-positive/trop-2-negative; red for EpCAM-negative/trop-2-positive; green for moderate expression of both EpCAM and Trop-2; and black for tissues negative for both EpCAM and Trop-2. Expression levels are measured in normalized protein-coding transcripts per million (nTPM).

Although the matriptase-EpCAM axis and the matriptase-Trop-2 axis exhibit a great many similarities, there is an interesting lack of redundancy apparent when expression patterns in human tissues are compared (Figure 5). Analysis of mRNA data from 55 human tissue types available in the Human Protein Atlas (https://www.proteinatlas.org/), showed that both matriptase and E-Cadherin were consistently detected in nearly all tissue specimens containing epithelium. In contrast, the expression of EpCAM and Trop-2 is more selective, with these related genes sometimes exhibiting a mutually exclusive expression pattern in certain tissues. In tissues such as esophagus, vagina, skin, urinary bladder, and tonsil, characterized by their barrier functions and resistance to stretching due to their composition of stratified squamous epithelium or related transitional epithelium (Figure 5), Trop-2 was detected at relatively high levels, whereas EpCAM expression was negligible. A reversed expression pattern, characterized by relatively high EpCAM levels and negligible Trop-2 expression, was observed in certain other tissues (Figure 5). These include certain tissues of the gastrointestinal (GI) tract such as the duodenum, small intestine, colon, rectum, and appendix, all of which feature simple columnar epithelium with microvilli on the apical surface for absorption. Endocrine glands such as the thyroid gland, parathyroid gland, adrenal gland, and pituitary gland, exhibit the same pattern, although notably, EpCAM expression in the adrenal gland was very low. This pattern is also seen in tissues with columnar epithelial cells featuring apical membrane projections, such as epididymal cells, which have long stereocilia to resorb fluid and facilitate sperm movement, gallbladder cells, which possess microvilli to absorb and concentrate bile, and fallopian tube cells, which are either ciliated to propel eggs or have microvilli.

In summary, these somewhat mutually exclusive expression profiles suggest that, despite their closely related roles as cell-cell adhesion molecules with highly similar physiological functions, Trop-2 and EpCAM may independently contribute to or be associated with distinct epithelial differentiation, leading to the morphological and functional divergence of the epithelium. Furthermore, matriptase, through its close enzyme-substrate relationships with both EpCAM and Trop-2 may also play a role in driving epithelial morphological and functional divergence in a distinct yet closely related manner. Notably, given the difference in the range of the Y-axis, 200 nTPM *versus* 800–1,000 nTPM, the overall expression level of matriptase is much lower than Trop-2 and EpCAM, consistent with their enzyme-substrate relationships.

Unlike the divergent expression patterns observed when comparing stratified squamous epithelium and simple columnar epithelium, EpCAM and Trop-2 can be found expressed together, typically at moderate to low levels, in a variety of tissues (Figure 5). This includes tissues such as the exocrine glands including the breast, prostate, salivary gland, and pancreas, all containing both glandular and ductal components. The same is true of some tissues with tubular glands such as the seminal vesicle and endometrium, which are again comprised of both ducts and glands. Tissues with multiple epithelial types such as the cervix, lung, and stomach, also exhibit a similar expression pattern. The moderate expression levels observed may result from the coexistence of multiple different epithelial types within these tissues if the apparent mutually exclusive expression of EpCAM and Trop-2 noted above holds true. Alternatively, EpCAM and Trop-2 might be co-expressed at relatively low levels within individual epithelial cells.

### Epithelial cells can sense and respond to their chemical environment through a large dynamic range from minimal to extensive EpCAM and/or Trop-2 cleavage

**Figure 6.**
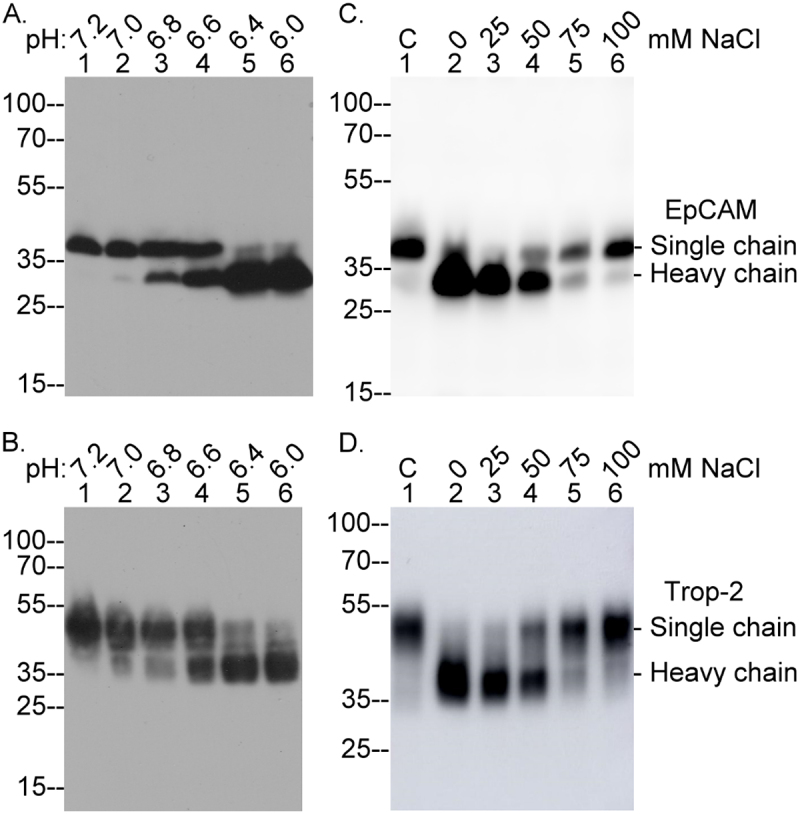
Rapid induction of different extents of EpCAM and Trop-2 cleavage by exposure to mildly acidic buffers and varying sodium chloride concentrationsT-47D breast cancer cells were transiently exposed to buffers with pH values of 7.2, 7.0, 6.8, 6.6, 6.4, and 6.0 (A and B, lanes 1–6) or to pH 6.0 buffers containing different sodium chloride concentrations of 0, 25, 50, 75, and 100 mM (C and D, lanes 2–6). Untreated cells served as a non-activation control (C and D, lane 1, labeled as ‘C’). Cell lysates were analyzed by immunoblot for EpCAM (A and C) and Trop-2 (B and D). The bands corresponding to single-chain EpCAM, heavy chain EpCAM, single-chain Trop-2, and heavy chain Trop-2 are indicated.

The precise temporal coupling of matriptase zymogen activation with EpCAM/Trop-2 cleavage (Figures 1, 2, and 3) suggests that the biological functions of the matriptase-EpCAM/Trop-2 axes are determined by the factors that induce matriptase zymogen activation, as well as the regulatory and functional implications of converting single-chain EpCAM/Trop-2 into their two-chain forms. Matriptase autoactivation requires cleavage at Arg-614, a process driven by the intrinsic activity of the matriptase zymogen itself rather than by other active proteases. Several factors can significantly modulate matriptase autoactivation, including chemicals such as chloride and molecules involved in regulating acidity and redox state. These components collectively form the constantly fluctuating yet tightly controlled chemical environment of cells [50]. The matriptase-EpCAM/Trop-2 axes likely functions to detect and translate changes in the cellular chemical environment into signals, ultimately leading to the production of two-chain EpCAM/Trop-2. This tight coupling ensures the rapid generation of two-chain EpCAM/Trop-2 in response to alterations in the cellular microenvironment. Importantly, the broad range of the extent of conversion to two-chain EpCAM/Trop-2 can be leveraged to reflect the degree of environmental changes. For instance, when cells were briefly exposed to buffers with varying pH levels, the production of two-chain EpCAM or Trop-2 increased progressively, ranging from minimal to extensive, as pH decreased (Figure 6(A,B)). Similarly, when different concentrations of sodium chloride were added to the acidic buffer, the levels of two-chain EpCAM/Trop-2 decreased in a dose-dependent manner, shifting from extensive to minimal (Figure 6(C,D)). This dynamic range for two-chain EpCAM/Trop-2 production in response to acidity and chloride concentration reflects the regulatory role of the matriptase-EpCAM/Trop-2 axes in sensing and adapting to the cellular chemical environment. Notably, in spite of analyzing equal amounts of cellular protein, the levels of the EpCAM heavy chain were often higher than those of single-chain EpCAM. This discrepancy may stem from differences in epitope exposure, which seem to vary somewhat between experiments.

### Matriptase-mediated cleavage doesn’t impact the function of EpCAM as a cell-cell adhesion molecule

**Figure 7.**
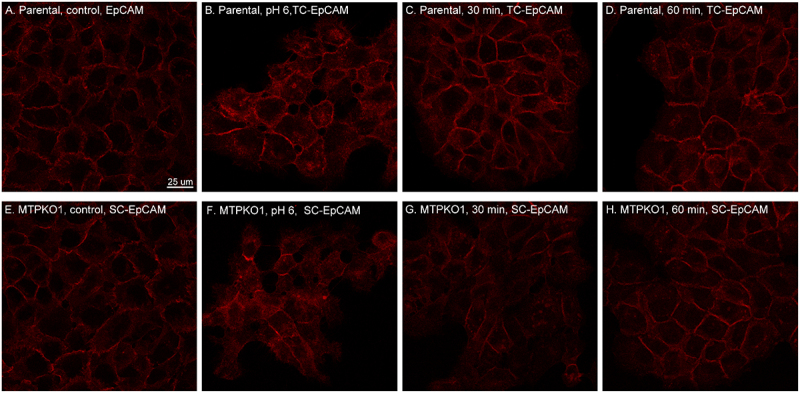
Rapid reformation of cell-cell junctions with EpCAM accumulation in parental and matriptase KO HaCaT cells following transient acid exposure. Parental HaCaT cells (A – D) and a matriptase KO variant (MTPKO1, E – H) were transiently exposed to a pH 6.0 buffer, then returned to regular culture medium and incubated for the indicated time points. The cells were analyzed by fluorescence confocal microscopy to detect EpCAM using an EpCAM mAb and F-actin using fluorescent dye-conjugated phalloidin (data not shown). TC stands for two-chain, and sc for single-chain. The scale bar sizes are shown.

**Figure 8.**
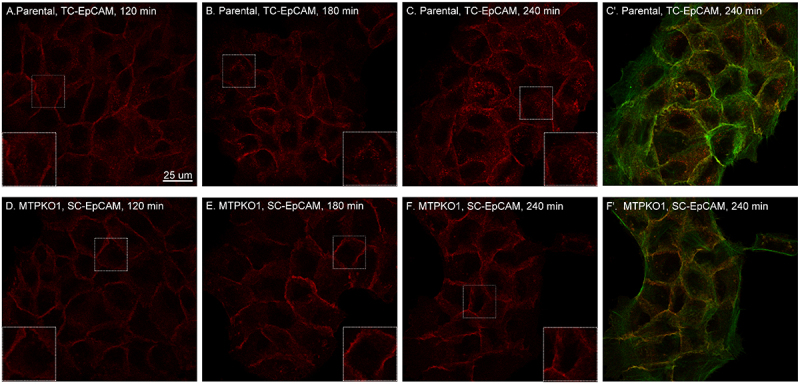
Rapid internalization of two-chain EpCAM, but not single-chain EpCAM. HaCaT human keratinocytes (A – C) and a matriptase knockout variant (MTPKO1, D – F) were briefly exposed to a pH 6.0 buffer, followed by a return to regular culture medium and incubation for the indicated times. The cells were analyzed by fluorescent confocal microscopy to detect EpCAM in red using an EpCAM mAb and F-actin in green using fluorescent dye-conjugated phalloidin. Merged images showing both EpCAM and F-actin are presented in panels C’ and F.’ Scale bar sizes are provided, and enlarged views for clarity and detail are shown in inserts taken from the regions marked with dotted squares. TC stands for two-chain, and sc for single-chain.

The functional role of the matriptase-EpCAM axis was further explored, with a focus on how cleavage affects EpCAM’s function as a cell-cell adhesion molecule and the fate of two-chain EpCAM. Both aspects can be assessed by monitoring the dynamic redistribution of EpCAM during the reestablishment of cell-cell adhesion. This process occurs after transient acid exposure, which stretches and weakens cell-cell interactions and was used to trigger matriptase zymogen activation and EpCAM cleavage. Using immunofluoresce confocal microscopy, strong EpCAM staining was observed at the cell-cell interfaces of parental HaCaT cells, with significant accumulation at these junctions (Figure 7(A)). In areas with less EpCAM accumulation, the staining appeared relatively dim and displayed various patterns, including a generally diffuse signal, dots of differing sizes and intensities, or localization in filopodia-like structures (Figure 7(A)). As a prominent cell-cell adhesion molecule, this staining pattern is expected and is consistent with the dynamic redistribution of EpCAM between cell junctions and the unbound cell surface, facilitating the intercellular interactions and adjustments critical for maintaining epithelial clusters. A similar staining pattern was observed in the matriptase KO variant (Figure 7(E)), although the KO cells are noticeably larger compared to the of parental cells. As shown in Figure 1, EpCAM exists in its single-chain form in both HaCaT cells and the MTPKO1 variant. However, transient acid exposure can convert EpCAM to its two-chain form in the parental cells but not in MTPKO1 cells. Acid exposure caused redistribution of EpCAM resulting in a more diffuse pattern and an increased accumulation at some cellular interfaces in both parental and KO cells (Figure 7(B,F)). When these cells were returned to culture medium, parental and KO cells underwent continuous changes in the subcellular distribution of EpCAM. Representative EpCAM staining at various time points is shown in Figures 7 and 8. Between 30 and 60 minutes after being returned to culture medium, both parental and KO cells exhibited rapid EpCAM translocation to cell-cell junctions and changes in cellular morphology. The re-formation of cell-cell junctions did, however, occur more rapidly in the parental cells compared to the KO variant (Figure 7). By 30 minutes, in a significant proportion of the parental cells EpCAM had translocated and accumulated at cell-cell junctions (Figure 7(C)). At 60 minutes, this accumulation became less intense and uniform, with a more diffuse staining pattern appearing (Figure 7(D)). In MTPKO1 cells, EpCAM recovery at the cell-cell junctions was observed 60 minutes after returning to normal medium, with a transitional phase at around 30 minutes during which the cells displayed altered morphology with less abundant EpCAM accumulation at the cell-cell junctions (Figure 7(F,G)). In spite of the slower kinetics in the KO cells, the patterns of translocation and accumulation of EpCAM at cell-cell junctions were similar between the two cell lines. This similarity suggests that there is no significant difference in the functionality of single-chain EpCAM in KO cells compared to two-chain EpCAM in the parental cells. Both forms demonstrate comparable abilities for homotypic interactions and oligomerization mediated by EpCAM’s extracellular domains, as well as for connecting to F-actin via interactions between EpCAM’s intracellular domain and alpha-actinin [59]. These functions are essential for EpCAM’s role as a cell-cell adhesion molecule.

### The matriptase-mediated cleavage of EpCAM is succeeded by its internalization, removal from the cell surface, and disassociation from F-actin

Although two-chain EpCAM and single-chain EpCAM exhibit similar behavior during the early stages of recovery from acid-mediated morphological changes (Figure 7), two-chain EpCAM, unlike its single-chain counterpart, begins to undergo internalization, albeit to a small extent one hour after returning to the regular culture medium. In parental cells, internalized two-chain EpCAM appears as small intracellular dots, which progressively increase in number and signal intensity in more of the cells over time (Figure 8(A–C)). In contrast, this gradual increase in intracellular staining is not observed in the matriptase-deficient variant cells, indicating that single-chain EpCAM does not undergo internalization (Figure 8(D–F)). Notably, as expected, the internalized two-chain EpCAM present in small vesicles does not co-localize with F-actin, appearing as distinct red dots in the merged image with F-actin (Figure 8(C’)). This lack of colocalization highlights the disconnection of internalized two-chain EpCAM from F-actin. In contrast, the two-chain EpCAM in parental cells, that has not been internalized, along with single-chain EpCAM in the matriptase-deficient variant cells, remains colocalized with F-actin, particularly at cell-cell contacts or in larger patches, appearing yellowish in the merged images (Figure 8(C’,F’)). The internalization and loss of association with F-actin, facilitated by matriptase-mediated cleavage, combined with the matriptase zymogen activation mechanisms (Figures 1 and 6), establish the functional framework of the matriptase-EpCAM axis. Within this framework, EpCAM-mediated cell-cell adhesion is disrupted through the internalization, removal, and dissociation of two-chain EpCAM from F-actin. In the broader context of EpCAM cleavage, this disruption can occur in specific cells, at distinct surface locations, and to various extents.

### Two-chain Trop-2 undergoes internalization more rapidly than two-chain EpCAM

**Figure 9.**
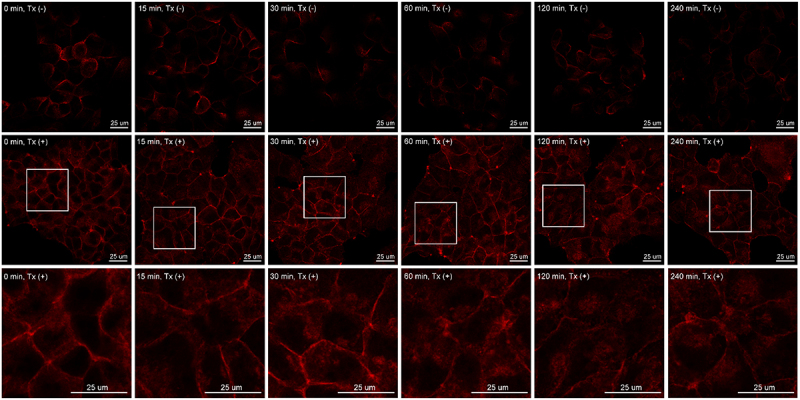
Rapid internalization of two-chain Trop-2. T-47D human breast cancer cells were briefly treated with a pH 6.0 buffer for 15 minutes, followed by incubation in regular culture medium for the indicated times. Trop-2 localization was analyzed using fluorescent confocal microscopy with an Trop-2 mAb under non-permeabilizing conditions [upper panels, Tx(-)] and permeabilizing conditions [middle and lower panels, Tx(+)]. Enlarged views for clarity are provided in the lower panels, highlighting regions marked by white squares in the middle panels. Scale bars are included for reference.

Given the close relationship between EpCAM and Trop-2, we next examined the impact of acid-induced cleavage on Trop-2 dynamics. Although both two-chain Trop-2 and two-chain EpCAM rapidly accumulate at the cellular interface and promote cell-cell adhesion, they differ in their internalization behavior after acid treatment and return to standard culture conditions. Notably, two-chain Trop-2 internalizes significantly earlier than two-chain EpCAM. A complicating factor is the presence of a Trop-2 fragment slightly smaller than 10-kDa, found within intracellular vesicle-like structures in a subset of cells independent of matriptase expression. This fragment interferes with detection of internalized two-chain Trop-2, particularly in HaCaT cells where its expression is relatively elevated. In T-47D cells, its minimal abundance allows clearer assessment of internalization. Further characterization of the 10-kDa Trop-2 fragment is provided in the Supplemental Materials (Figures S1 and S2). To circumvent this problem and simplify analysis, we switched to T-47D breast cancer cells as a model to clearly demonstrate the rapid internalization of two-chain Trop-2 since the level of the 10-kDa Trop-2 fragment is much lower in these cells (Figure 9).

Following transient exposure to a mildly acidic buffer, T-47D breast cancer cells were returned to their regular culture medium and incubated at 37°C for 15 min, 30 min, 60 min, 120 min, or 240 min. The dynamic changes in the subcellular localization of Trop-2 were then analyzed by indirect immunofluorescence staining. As shown in Figure 9 (upper panels) under non-permeabilizing conditions, the strong cell surface staining of Trop-2 at the initial time point (*T* = 0) began to diminish 30 minutes after the cells were returned to the regular medium, and the signal was almost completely lost by 240 minutes. Under permeabilizing conditions using Triton X-100 (Tx) (Figure 9, middle panels), the overall levels of cellular Trop-2 appeared relatively unchanged. However, peripheral Trop-2 staining disappeared over time, mirroring the changes observed in non-permeabilized cells. At higher magnification (Figure 9, lower panels), the loss of surface staining can be seen to be accompanied by the emergence of intracellular punctate staining. These changes in immunofluorescent staining suggest that Trop-2 is rapidly internalized from the cell surface following its conversion from the single-chain to the two-chain form.

### EpCAM and Trop-2 are degraded following matriptase-mediated cleavage and internalization, and are subsequently replenished through de novo protein synthesis

**Figure 10.**
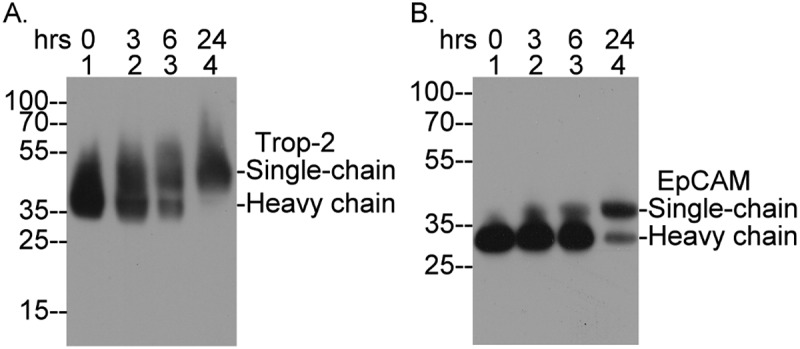
Degradation of internalized two-chain EpCAM and Trop-2 and the de novo synthesis of their single-chain counterparts. T-47D human breast cancer cells were exposed to a pH 6.0 buffer for 15 minutes, then returned to regular culture medium for the indicated times. Cell lysates were analyzed by immunoblotting for Trop-2 (a) and EpCAM (b). The single-chain and heavy chain forms of EpCAM and Trop-2 are labeled.

The fate of EpCAM and Trop-2 following matriptase-mediated cleavage and internalization was next investigated by tracking the disappearance of two-chain EpCAM and Trop-2 at 3, 6, and 24 hours after reintroduction to culture medium (Figure 10). Consistent with the observed rapid internalization (Figure 9), a marked degradation and significant reduction in the levels of two-chain Trop-2 was evident within 3 hours (Figure 10(A)). The degradation progressed further, culminating in the complete disappearance of two-chain Trop-2 by 24 hours. Notably, single-chain Trop-2 was clearly detected at 6 hours, though it may have started to appear as early as 3 hours. By 24 hours, significant levels of single-chain Trop-2 were again detectable. These findings demonstrate that Trop-2 undergoes rapid turnover through intracellular degradation and is replenished via de novo protein synthesis following induced cleavage. Although the fate of EpCAM closely resembled that of Trop-2, its degradation commenced only after 6 hours of reintroduction to the culture medium and was nearly complete by 24 hours (Figure 10(B)). This delayed degradation corresponds to the longer time required for the internalization of two-chain EpCAM (Figure 8). De novo synthesis of EpCAM began as early as 3 hours, with substantial accumulation observed at 24 hours (Figure 10(B)). The newly synthesized Trop-2 and EpCAM not only compensate for their loss but also restore cell-cell adhesion through their intrinsic homotypic interactions.

The molecular events triggered by matriptase-mediated cleavage, including internalization, degradation, and de novo protein synthesis, provide cells with a mechanism to dynamically dissociate and rebuild EpCAM- and Trop-2-mediated cell-cell adhesions in a self-regulated manner. Similar self-regulated processes also occur with matriptase, where zymogen activation is followed by HAI-1-mediated inhibition of the nascent active matriptase, shedding of the matriptase-HAI-1 complex, and de novo synthesis of the matriptase zymogen [49]. With these closely integrated molecular events, the TTSP and the two CAMs can act in concert to execute their biological functions with the matriptase-EpCAM axis predominantly in simple epithelia and the matriptase-Trop-2 axis predominantly in stratified squamous epithelia. The protease-CAM axis represents novel mechanism by which cell-cell adhesion can be modulated in a highly adaptable and rapidly responsive manner to changes in cellular microenvironments, including acidity, redox state, and salt concentration.

### The matriptase-CAM axis is crucial in the maintenance of epithelial integrity

**Figure 11.**
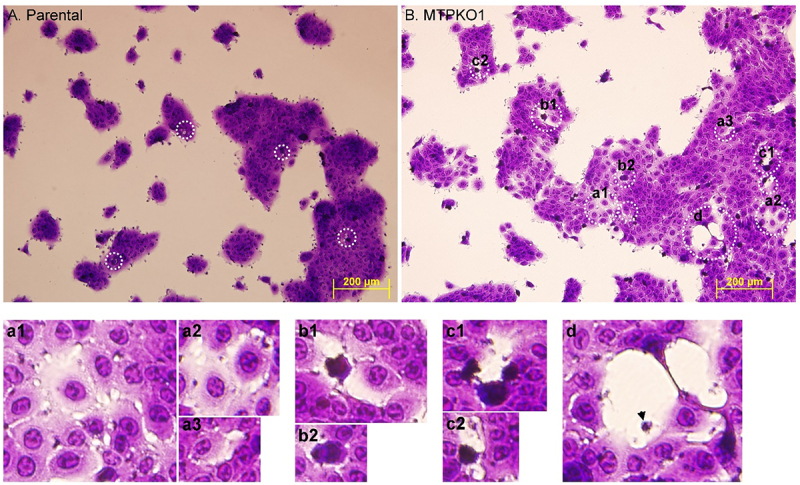
Matriptase-deficient HaCaT keratinocytes form porous epithelial colonies. Crystal violet staining was used to perform morphological analyses on parental HaCaT human keratinocytes (A) and a matriptase knockout variant (B) to evaluate the effect of matriptase deletion on cell packing. Areas with intercellular gaps and holes are marked by white dashed circles. Some of these regions in the matriptase knockout variant are shown at higher magnifications for clarity (a – d). A cell debris fragment is indicated by an arrowhead (d).

The role of the protease-CAM axis in the modulation of cell-cell adhesion was next characterized in a HaCaT variant, in which the impact of matriptase deletion on cellular and colony morphology were easily observed (Figure 11) along with the disruption of the cleavage and subsequent internalization of EpCAM and Trop-2 (Figures 8 and 9). Crystal violet staining revealed that parental HaCaT cells were tightly packed together, regardless of colony sizes, with fused boundaries that were difficult to distinguish at low magnification. In contrast, in addition to an expanded morphology, most colonies of MTPKO1 cells exhibited a porous structure with intercellular gaps and holes of varying sizes (Figure 11(B), indicated by dashed white circles). At higher magnifications, some cells exhibited even more expanded morphologies, with spreading membrane protrusions, and discontinuous connections with neighboring cells displayed small intercellular gaps, while maintaining round nuclei similar to the adjacent cells (Figure 11(a1–a3)). Larger intercellular gaps and holes were found among irregularly shaped, densely stained cells (Figure 11(b1,b2,c1, and c2)). In some epithelial clusters, extensive gaps were observed where cells were detaching, with only thin, highly stretched plasma membrane strands remaining as connections (Figure 11(d)). While intercellular spaces or holes were also observed in parental cells (Figure 11(A), dashed white circles), they were significantly smaller and less frequent than in the matriptase-deficient counterparts.

**Figure 12.**
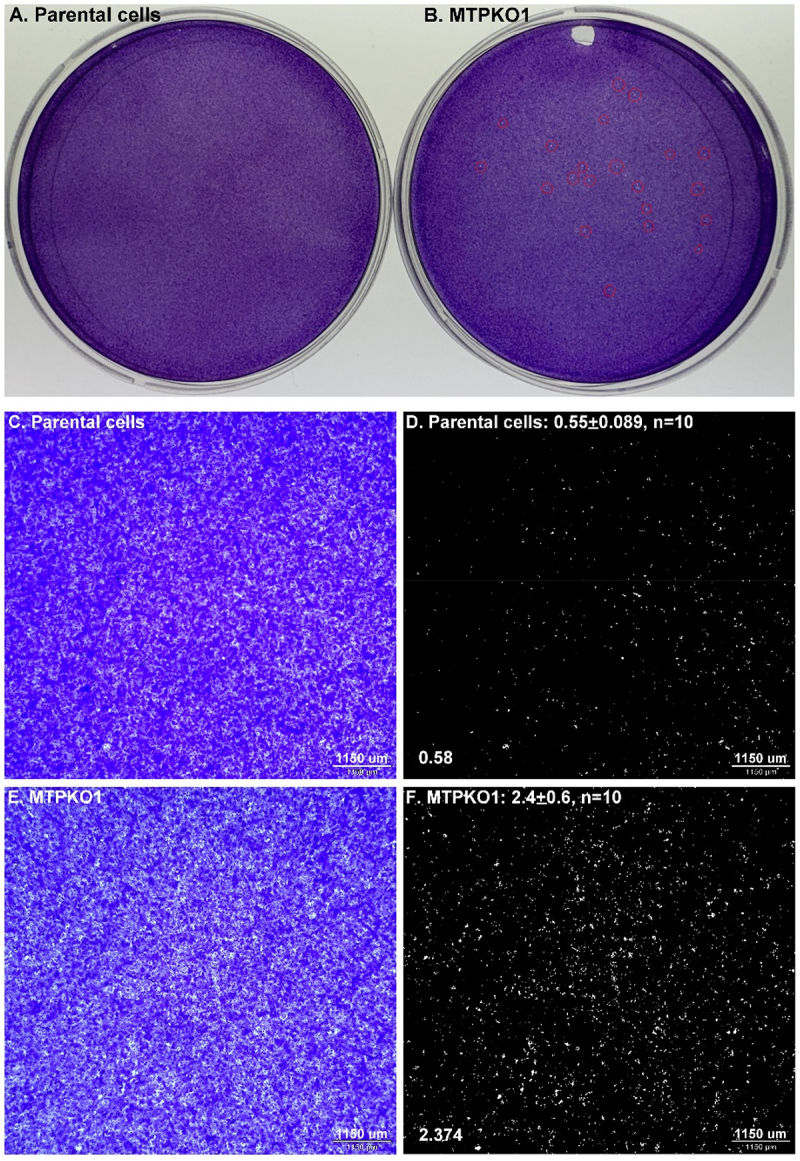
Matriptase knockout (KO) variants exhibit fourfold higher epithelial porosity compared to parental cells. Parental HaCaT human keratinocytes (A, C, D) and a matriptase KO variant, MTPKO1 (B, E, F), were cultured to near confluency in 60 mm dishes. Crystal violet staining was used to assess epithelial layer porosity and evaluate the impact of matriptase deletion. Visible gaps in the KO cells are highlighted with red dashed circles (b). Microscopic images (C and E) were converted to binary format (D and F) using ImageJ for quantitative porosity analysis. A total of ten images (*n* = 10) per cell type were analyzed. The ratio of intercellular gaps to the total epithelial area is displayed in the lower left corner of the representative images with group averages shown at the top (D and F).

The porosity of the epithelial layer was compared in a quantitative fashion between parental HaCaT cells and the KO variants grown to near confluency. After staining with crystal violet, numerous gaps were apparent in the KO variants, while such gaps were barely detectable in the parental cells (Figure 12(B), highlighted by red dashed circles). Microscopic analysis further revealed that even in areas where cells appeared more uniformly packed, the intercellular gaps were significantly more frequent in the KO variants compared to the parental counterparts (Figure 12(C,E)). By converting the images to binary format to differentiate cells from gaps, the ratio of intercellular gaps to the total epithelial area was found to be approximately four times higher in the KO cells than in the parental cells (2.373/0.58 = 4.09). This ratio was calculated from ten randomly selected regions where cells were relatively uniformly distributed. Representative images with ratios close to the group average are shown in Figure 12(D,F). The porous epithelial layers formed in MTPKO1 cells suggest that the dynamic modulation of cell-cell adhesion via matriptase-mediated cleavage, internalization and protein syntheses of EpCAM and Trop-2 is critical in maintaining a tightly connected epithelial layer.

### Matriptase zymogen activation occurs with cell death and prior to cell division

The increased presence of intercellular gaps and holes (Figures 11 and 12) suggests that the loss of matriptase impairs some cellular processes dependent on the finely tuned disassembly and reassembly of cell-cell adhesions, such as in cell extrusion and cell division. Cell extrusion is essential to facilitate seamless removal of unwanted cells from the epithelial layer and can be triggered by several stimuli, including apoptosis [17,60]. Disruptions in this process may lead to the formation of gaps within the epithelial layer. Dynamic modulation of cell-cell adhesions is also critical during cell division, which requires orchestrated changes in cell shape, adhesion properties, and cytoskeletal architecture, largely regulated by cell adhesion molecules, for accurate chromosome segregation, successful cytokinesis, and reformation of cell-cell adhesion with neighboring cells [61,62].

**Figure 13.**
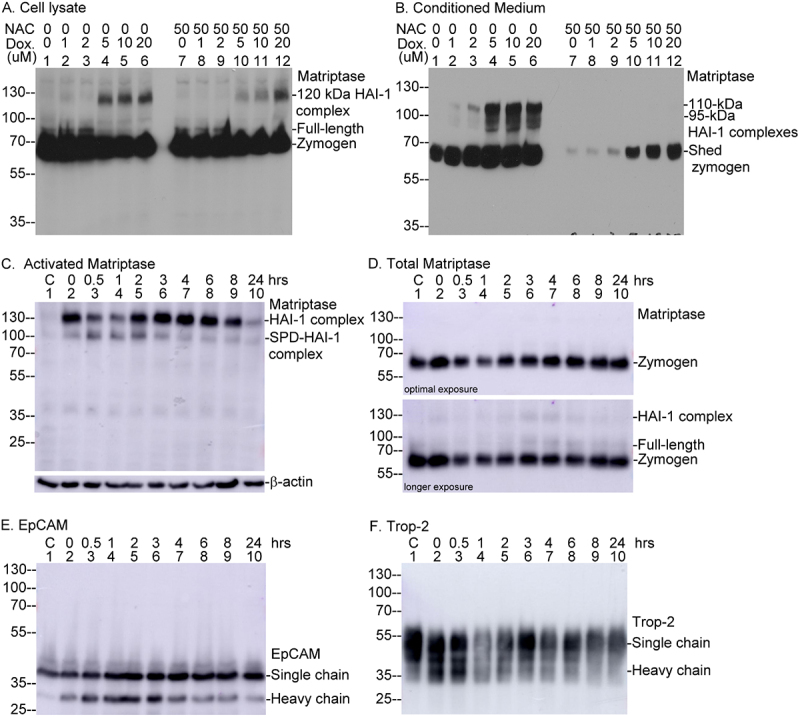
Activation of the matriptase-CAM axis occurs concurrent with apoptosis and during the early stages of cell division. Panels A and B: MCF-7 human breast cancer cells were treated overnight with doxorubicin at the indicated concentrations, with or without 50 µM N-acetylcysteine (NAC). Cell lysates (A) and conditioned media (B) were subjected to immunoblot analysis to detect various forms of matriptase, as labeled. Panels C – F: HaCaT human keratinocytes were synchronized via a double thymidine block and then released into normal growth medium for the indicated time periods. Cell lysates, including a pre-synchronization sample (lane 1), were analyzed by western blot for activated matriptase (C), total matriptase (D), EpCAM (E), Trop-2 (F), and β-actin as a loading control (C, lower panel). Detected forms of EpCAM, Trop-2, and matriptase, including the matriptase serine protease domain (SPD) in complex with HAI-1, are indicated.

If the protease-CAM axis is involved in apoptosis-mediated cell extrusion, one might expect the induction of matriptase zymogen activation by apoptosis-inducing agents such as doxorubicin, a chemotherapeutic drug known to cause oxidative stress and cell death. Indeed, treatment of cells with concentrations above 5 µM, resulted in the appearance of the 120-kDa activated matriptase-HAI-1 complex in cell lysates (Figure 13(A), lanes 4–6) and the degraded forms of the complex detected as bands of 110- and 95-kDa were also found in conditioned media from the cells (Figure 13(B), lanes 4–6). Given that oxidative stress underlies both doxorubicin-induced apoptosis [63,64] and matriptase activation [50], the addition of 50 µM N-acetylcysteine (NAC) significantly attenuated matriptase activation and shedding (Figure 13(A,B), lanes 7–12).

Apoptosis-induced matriptase zymogen activation was also observed when HaCaT cells underwent prolonged exposure to high-dose thymidine (Figure 13(C), lane 2 versus lane 1), which was used to synchronize the cells [65]. To explore the involvement of the matriptase-EpCAM/Trop-2 axes in cell division, we monitored matriptase zymogen activation following release from a double thymidine block, synchronizing cells at the entry to S phase. Upon return to normal culture conditions, both matriptase zymogen and the activated forms declined rapidly within the first hour (Figure 13(C,D), lanes 3–4), likely reflecting accelerated shedding [56]. By the second hour, levels of both forms increased sharply, indicative of rapid de novo protein synthesis and zymogen activation. Matriptase levels remained stably elevated for the next four hours (Figure 13(C,D), lanes 5–8), suggesting an equilibrium between synthesis, zymogen activation, and shedding. After eight hours, activated matriptase levels slightly declined (Figure 13(C), lane 9) and returned to baseline by 24 hours, comparable to the pre-synchronization state (Figure 13(C), lanes 10 versus lanes 1). These dynamic changes in matriptase activation closely correlate with cell cycle progression, with elevated activation aligning with S-phase entry, coinciding with the onset of cell-cell adhesion remodeling. Notably, despite the increase in matriptase-HAI-1 complexes, the overall extent of matriptase zymogen activation induced by the double thymidine block and at the S-phase of dividing cells remained relatively low, as evidenced by the requirement for prolonged immunoblot exposure (Figure 13(D), lower panel *versus* upper panel) and the low activated-to-total matriptase ratio assessed with a total matriptase antibody (Figure 13(D), lower panel, HAI-1 complex versus zymogen). Furthermore, the low activated-to-total matriptase ratio can be attributed not only to the relatively low level of matriptase zymogen but also to rapid matriptase shedding. The low ratio also reflects only a small proportion of HaCaT cells undergoing apoptosis caused by thymidine treatment and the slight difference in the time for S-phase entry even among the synchronized cell population.

The cleavage of EpCAM and Trop-2 was also induced by double thymidine block, as evidenced by an increase in the level of two-chain EpCAM and Trop-2 (Figure 13(E,F), lanes 2). In contrast to the rapid shedding of matriptase, EpCAM and Trop-2 are internalized following the matriptase-mediated cleavage. Notably two-chain Trop-2 was internalized right after cleavage with more than half of two-chain Trop-2 being internalized and digested one hour after cleavage (Figures 9 and 10). In contrast, it takes much longer for two-chain EpCAM to be internalized and digested (Figures 8 and 10). With the difference in time frame for internalization, digestion, and de novo protein synthesis, two-chain EpCAM was observed to slightly increase 0.5 hour after release from double thymidine block (Figure 12(E), lane 3 versus lane 2), remained at the elevated level for 3 hours (Figure 12(E), lanes 3–6), and gradually decrease thereafter (Figure 13(E), lanes 7–10). Two-chain Trop-2 remained at a high level 0.5 hour after release from double thymidine block (Figure 13(F), lane 3) and rapidly returned to and remained at a low level over the course of 24-hour (Figure 13(F), lanes 4–10). The level of single-chain Trop-2 fluctuated significantly over the course of 24- hours, likely due to the dynamic interactions of internalization, digestion, and de novo protein synthesis, combined with the variation in the S-phase entry among different cells. The concurrent matriptase zymogen activation and cleavage of EpCAM and Trop-2 suggests that the protease-CAM axis is activated in response to the double thymidine block and the entry to S-phase, providing strong evidence for the role played by the matriptase-CAM axis in cell extrusion and division.

### Compromise of the matriptase-EpCAM/Trop-2 axes reduces the number of extruded cells

**Figure 14.**
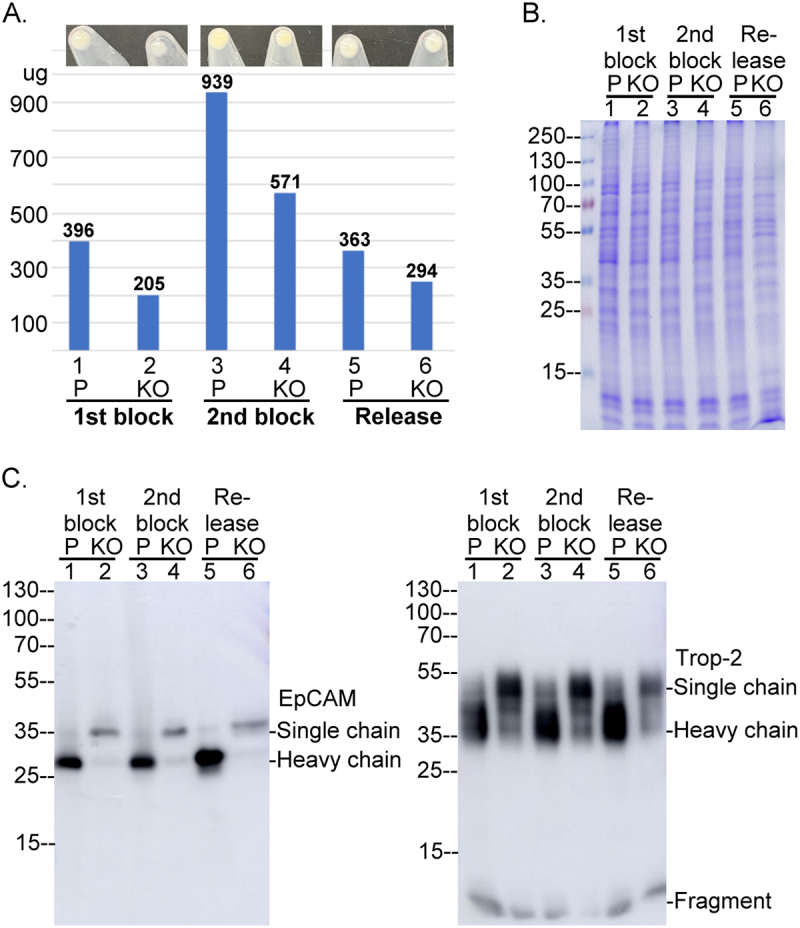
Targeted deletion of matriptase reduces cell debris shedding. Parental HaCaT human keratinocytes (P) and a matriptase knockout variant (KO) were subjected to double thymidine block for cell synchronization. Conditioned media were collected at key stages, first block, second block, and release, and insoluble materials were harvested. These materials were analyzed for abundance and total protein content (A), protein profiles via SDS-PAGE followed by coomassie brilliant blue staining (B), and the presence and forms of EpCAM and Trop-2, through immunoblotting (C).

To assess the effect of matriptase deletion on cell extrusion, insoluble material, primarily composed of coarse cell debris, was collected from the conditioned media of both parental HaCaT cells and matriptase KO variants at three key stages of the thymidine block protocol: the first block (16 hours), second block (16 hours), and release phase (24 hours). Although no clear morphological differences were observed between parental and KO variants in the insoluble material, the quantity of this material present in the conditioned medium was significantly different. At every stage, based on three independent experiments, the parental cells produced considerably more insoluble material than matriptase KO cells, (a representative result shown in Figure 14(A) (upper panels)), a finding corroborated by the total amount of RIPA-soluble protein extracted from these materials (Figure 14(A)). Coomassie Brilliant Blue (CBB) staining revealed nearly identical protein patterns, indicating that the overall composition of the insoluble material was grossly similar regardless of matriptase status (Figure 14(B)). However, western blot analysis showed that in debris from the parental cells, EpCAM and Trop-2 were predominantly present in their cleaved, two-chain forms across multiple stages (Figure 14(C), lanes 1, 3, and 5). This pattern suggests that cleavage of these CAMs occurs extensively in cells that are about to be extruded. In contrast, debris from the matriptase KO variants contained mostly single-chain forms of EpCAM and Trop-2 (Figure 14(C), lanes 2, 4, and 6), indicating that matriptase is necessary for efficient cleavage of EpCAM and Trop-2 in the process of shedding cell debris. Importantly, the reduction in cleaved EpCAM and Trop-2 in KO cells (Figure 14(C)) coincides with a decrease in shed debris (Figure 14(A)), suggesting that matriptase-mediated processing of these adhesion molecules plays a crucial role in preparing cells for extrusion. This conclusion is further supported by the marked increase in intercellular gaps and holes observed upon matriptase deletion (Figure 11), a phenotype characteristic of disrupted cell extrusion. Collectively, these findings support a model in which the matriptase-EpCAM/Trop-2 axes are activated in cells destined for extrusion, such as apoptotic cells induced by prolonged thymidine exposure, facilitating their detachment and removal from the epithelial sheet.

Notably, in addition to the lack of cleavage, the total level of EpCAM in the cell debris was consistently far less in the debris collected from the KO variant than from the parental cells (Figure 14(C), left panel). This is in contrast to the comparable levels of Trop-2 in both the parental and the variant cells (Figure 14(C), right panel). The selectively lower level of EpCAM suggests that those apoptotic matriptase KO cells destined for cell extrusion might express lower level of EpCAM.

### The matriptase-EpCAM/Trop-2 axes promote mitotic cell rounding but not post-cytokinetic re-adhesion

**Figure 15.**
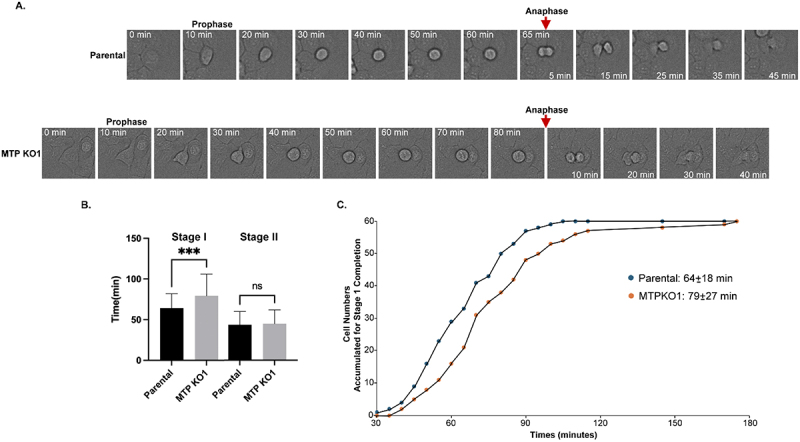
Matriptase loss extends mitotic cell rounding but does not affect post-cytokinetic re-adhesion. Live-cell imaging was used to monitor the division of 60 individual parental HaCaT human keratinocytes and their matriptase-knockout counterpart, MTPKO1. The mitotic process was divided into two phases: stage I (approximately spanning from prophase to metaphase) and Stage ii (from anaphase to cell reintegration). Representative image sequences are shown in panel A. The average durations of each stage were measured across the 60 cells and statistically compared between parental and MTPKO1 cells using unpaired t-test (panel B), where ***p < .001 and ‘ns’ denotes no significant difference. Time distributions for completing Stage I were further analyzed using an inverse Kaplan–meier-like curve (panel C).

To assess the role of matriptase-mediated EpCAM and Trop-2 cleavage in cell division, live-cell imaging was conducted over 18 hours with images collected at 10-minute intervals. Dividing cells were observed to undergo rapid morphological changes that impacted intercellular adhesion. For analysis, the division process was arbitrarily divided into two phases: Stage I, defined as starting with abrupt morphological changes such as cell rounding and ending around metaphase; and Stage II, encompassing anaphase, telophase, cytokinesis, and reintegration of daughter cells with neighboring cells. Representative images from both parental and knockout (KO) cells are shown in Figure 15(A). When cells were captured during telophase, the 10-minute imaging interval was split, assigning the first half to Stage I and the second to Stage II (Figure 15(A), upper panels). Analysis of 60 dividing cells showed that parental cells completed Stage I in an average of 64 ± 18 minutes, whereas KO cells required significantly more time, averaging 79 ± 27 minutes (Figure 15(B)). However, both cell types required similar time to complete Stage II, averaging approximately 45 minutes. Stage I completion times varied considerably among the 60 parental cells, ranging from 30 to 105 minutes, as depicted in an inverse Kaplan-Meier-like curve (Figure 15(C)). KO cells showed even greater variability, with Stage I durations ranging from 40 to 175 minutes. The prolonged Stage I in KO cells suggests that matriptase-mediated cleavage of EpCAM and Trop-2 facilitates mitotic cell rounding by weakening intercellular adhesions, while still preserving partial connectivity with surrounding cells. This interpretation is supported by increased matriptase zymogen activation and cleavage of EpCAM and Trop-2 shortly after release from double thymidine block in parental HaCaT cells (Figure 13). In contrast, the matriptase-CAM axis appears to play a lesser role in Stage II, during which daughter cells spread and reestablish adhesions with neighboring cells.

### Matriptase deficiency disrupts the cohesive structure and collective migratory capacity of keratinocytes

**Figure 16.**
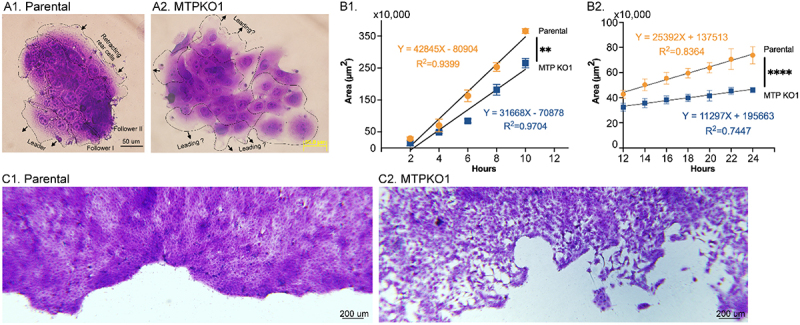
Matriptase-deficient HaCaT keratinocytes show reduced collective migration and disrupted structural cohesion. Parental HaCaT human keratinocytes (A1) and the matriptase knockout variant MTPKO1 (A2) were stained with crystal violet for morphological analysis. Representative migrating colonies of each cell type were selected, highlighting the well-organized leader-follower arrangement in the parental cells compared to the disorganized structure of MTPKO1 cells. The layered architecture and inferred migration directions are indicated. Both cell types underwent scratch wound healing assays with live-cell imaging (B1 and B2). Migration rates were assessed by measuring the repopulation of the wounded area during two-hour intervals: 2–10 hours (B1) and 12–24 hours (B2). The migration rates were calculated through linear regression analysis and compared between parental and MTPKO1 using GraphPad Prism 10 and F-test, where **p < .01 and ****p < .0001. Crystal violet staining was used to compare the morphology of parental (C1) and knockout (C2) cells five days post-scratch.

The collective migration of epithelial cell colonies is another situation in which cell-cell adhesion must be dynamically modulated to maintain coordinated movement, mechanical communication, and leader-follower dynamics within the cell group [66,67]. Crystal violet staining revealed that HaCaT parental cell colonies exhibited various architectures as the result of their continuously migrating, colliding with one another, and merging into larger clusters. A subset of the parental cells maintains a well-organized architecture, exhibiting cohesive and directional migration. In a representative cluster (Figure 16(A1)), the leader cells displayed prominent membrane protrusions at the front and nuclei positioned at the rear. The direct follower cells also appeared stretched, maintaining discernible intercellular spaces, which were connected by thin, filopodia-like structures (Figure 16(A1), Follower I). In contrast, those follower cells without direct contact with the leader cells were densely packed, with a compact morphology and fused intercellular contacts. Cells in the rear showed retracted membrane protrusions with ruffled features, and their nuclei were positioned toward the front. These cells appeared to be colliding with those in the preceding layer, resulting in the lack of noticeable intercellular space, distinct from the spaces observed between the leading-edge cells and those in the second row (Figure 16(A1)). In contrast to the organized, layered structure seen in the migrating parental cell clusters (Figure 16(A1)), many migrating MTPKO1 clusters of similar size were packed together in a disorganized manner. As shown in a representative example (Figure 16(A2)), cells with stretched morphologies and membrane protrusions were found throughout the entire cluster, rather than being confined to specific regions. These cells seemed to attempt to function as leading cells but lacked followers. With numerous would-be leading cells scattered along the periphery, the interior cells were randomly packed without forming any ordered structure (Figure 16(A2)).

The disrupted organization and lack of a layered structure observed in MTPKO1 cells indicate that matriptase-dependent cleavage and internalization of EpCAM and Trop-2 are essential for HaCaT keratinocytes to assemble into tightly coordinated clusters. Without this structural organization, MTPKO1 cells appear to be less capable of migrating cohesively in a unified direction, unlike their parental counterparts. This hypothesis was subsequently tested and confirmed using the scratch wound healing assay, a well-established model for studying collective migration (Figure 16(B1,B2)) [68]. The migration rate was assessed based on the rate at which cells repopulated the wounded area over a 24-hour period, with live-cell microscopy capturing images at 2-hour intervals. Interestingly, both knockout and parental cells showed more rapid migration during the initial 12 hours, followed by a slower phase thereafter. However, the parental cells migrated significantly faster than MTPKO1 cells: by 35% during the first 12 hours (42,845 vs. 31,668 units) and by 125% during the second 12 hours (25,392 vs. 11,297 units). Additionally, in a wide wound model, parental cells remained compact and well-organized at the migrating front even after five days (Figure 16(C1)). In contrast, to the MTPKO1 cells which exhibited a progressively more disorganized and porous leading edge, with many cells detaching from the main sheet and migrating individually or in small clusters (Figure 16(C2)). These findings reinforce the conclusion that the dynamic regulation of cell-cell adhesion mediated by EpCAM and Trop-2 processing is critical for the collective migration of HaCaT keratinocytes.

## Discussion

**Figure 17.**
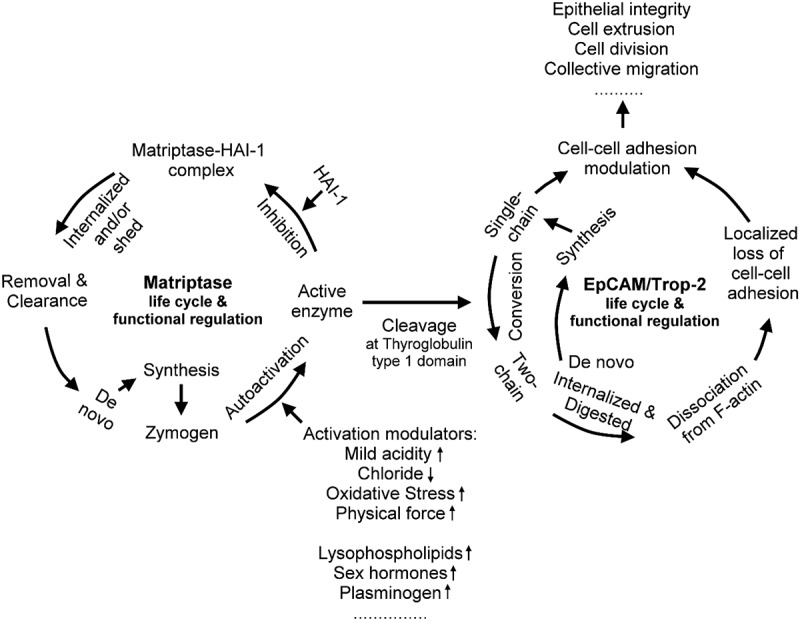
The matriptase – EpCAM/Trop-2 axis. A schematic representation of the life cycles and regulatory mechanisms of matriptase and EpCAM/Trop-2, which together constitute the protease-CAM axis via matriptase-mediated cleavage of EpCAM/Trop-2. Further details on the protease-CAM axis are provided in the discussion section.

The discovery and functional characterization of the matriptase-EpCAM axis and the matriptase-Trop-2 axis, two critical cell surface mechanisms governing dynamic modulation of intercellular adhesion essential for epithelial cell extrusion, division, and collective migration, began with establishing the enzyme-substrate relationship. This relationship is characterized by a tightly coordinated sequence of molecular events, dependent on anchorage at the lipid bilayer membrane to ensure their rapid and efficient interactions. The physiological relevance of this biochemical enzyme-substrate relationship is supported by the widespread co-expression of matriptase, EpCAM, and Trop-2 both *in vitro* and *in vivo*. In addition, matriptase plays an essential role in generating the two-chain forms of EpCAM and Trop-2. This has been demonstrated in physiologically relevant settings as well as through a matriptase activity induction model. The protease-CAM axis involves a series of tightly coordinated molecular events that proceed rapidly in a cascade-like manner once initiated. Based on our previous studies [35], matriptase is initially synthesized as an enzymatically inactive zymogen. Upon activation, the nascent active matriptase quickly cleaves its substrates or is inhibited by HAI-1, two simultaneous and competing processes that ensure limited substrate cleavage rather than the extensive digestion of EpCAM and/or Trop-2. Following inhibition by HAI-1, the resultant matriptase-HAI-1 complex is cleared from the cell by shedding of the complex, while new protein is synthesized to replenish matriptase levels. Interestingly, the matriptase-HAI-1 complex is found in a variety of human bodily fluids such as breast milk and semen. Zymogen matriptase is targeted to the basolateral surface of cells. The secretion of the matriptase-HAI-1 complex into extracellular fluids begins with its internalization from the basolateral membrane of epithelial cells. It is then transported via transcytosis and ultimately released into the lumen from the apical surface of lactating mammary epithelial cells or prostate epithelial cells. As shown in this study, EpCAM and Trop-2 are synthesized as mature single-chain proteins that spontaneously mediate cell-cell adhesion through homotypic interactions in either *trans* or *cis* configurations. Upon cleavage by newly active matriptase, these single-chain proteins are converted into two-chain forms, which are subsequently internalized and degraded with consequent loss of localized cell-cell adhesion. This is followed by de novo protein synthesis to replenish EpCAM or Trop-2 and rebuild cell-cell adhesions. Notably, despite their structural similarities, EpCAM and Trop-2 are expressed in a mutually exclusive manner between stratified squamous epithelia *versus* simple epithelia and exhibit a different lag time between the generation and internalization of the two-chain forms. The life cycles of matriptase and the two CAMs are illustrated in Figure 17.

The identification of EpCAM and Trop-2 as downstream matriptase substrates reveals that a key biological function of matriptase is its novel role in the regulation of cell-cell adhesion. This occurs through the localized internalization and subsequent degradation of two-chain EpCAM or Trop-2, followed by replenishment by the de novo synthesis of their single-chain forms. Such rapid cycles of dissociation and reforming of cell-cell adhesion enable individual epithelial cells to dynamically and responsively adjust their interactions with neighboring cells, influencing how epithelial cells pack together. Moreover, the wide range in the extent of EpCAM and Trop-2 cleavage that can rapidly occur, the protease-CAM axis provides epithelial cells with a highly regulable and adaptable mechanism for modulating cell-cell adhesion and epithelial cell packing. This highly adaptable mechanism, triggered by pericellular proteolysis, sets EpCAM and Trop-2 apart from E-cadherin. Unlike E-cadherin, which facilitates the formation of adherens junctions in a Ca^2+^ -dependent manner, EpCAM and Trop-2 mediate non-junctional cell-cell adhesion in a Ca^2+^ -independent manner. Although E-cadherin also undergoes proteolytic cleavage, it is primarily processed by members of the A Disintegrin And Metalloprotease (ADAM) family, leading to its shedding and the downregulation of adherens junctions [69–71]. However, the removal of E-cadherin from the cell surface, which is crucial for regulating cell-cell adhesion, predominantly occurs through endocytosis [72,73]. While both EpCAM/Trop-2 and E-cadherin rely on endocytosis for adhesion regulation, they differ in the mechanisms that trigger internalization and in how the internalized proteins influence cell-cell adhesion. Internalized E-cadherin can either be degraded in the lysosome or recycled back to the plasma membrane, allowing for the fine-tuning of adhesion dynamics and adherens junction assembly. In contrast, the internalized EpCAM and Trop-2 appear to be exclusively targeted for degradation. Since their degradation appears to be closely linked to *de novo* protein synthesis, it suggests a mechanism in which internalization or degradation induces new protein production. Thus, despite their similarities and differences, localized adhesion disruption is invariably followed by adhesion reformation.

Beyond the role of internalized CAMs in regulating adhesion, the mechanisms triggering internalization serve as a key distinguishing factor between the non-junctional CAMs and the junctional CAM. Disrupting the interactions of E-cadherin with the catenins, p120-catenin in particular, is believed to expose the endocytosic signal and functions as the most important step for E-cadherin endocytosis [74]. In contrast to the intracellular events that decouple E-cadherin from F-actin microfilaments, EpCAM and Trop-2 internalization is initiated by an extracellular mechanism. Specifically, matriptase mediates N-terminal cleavage within the thyroglobulin type 1 domain. This enzyme-dependent cleavage leads to the dissociation of EpCAM and Trop-2 from F-actin microfilaments. Although it is not clear how this N-terminal cleavage triggers internalization, the cleavage is expected to cause conformational changes through the generation of new N- and C-termini in the two fragments of EpCAM/Trop-2. These conformational changes could serve to directly or indirectly initiate endocytosis of two-chain EpCAM/Trop-2. Given the tightly coupling of matriptase zymogen activation and the cleavage of EpCAM and Trop-2, the mechanism triggering matriptase zymogen activation becomes the most important checkpoint for the functional regulation of the protease-CAM axis. Matriptase is initially synthesized as an enzymatically inactive zymogen, which can be converted to the active protease through an unconventional autoactivation process involving the zymogen-mediated cleavage at Arg-614 within the activation motif. A variety of apparently unrelated factors and cellular characteristics can modulate matriptase autoactivation in either a relatively ubiquitous or a cell-type selective manner. Matriptase autoactivation is sensitive to changes in pH, redox potential, and chloride concentration, major components of cellular chemical environments [50]. Furthermore, the autoactivation of matriptase, followed by cleavage of EpCAM and Trop-2, ensures tight regulation of cell adhesion molecule processing. This control applies to the extent of cleavage, as well as its timing and precise location. These features confer upon epithelial cells the ability to rapidly adjust their adhesion to and interaction with neighboring cells in response to the highly dynamic cellular chemical environments.

In addition to alterations in the cellular chemical environment, changes in cell-cell adhesion and junctions themselves can also influence matriptase autoactivation. Ultrasound-mediated microbubble cavitation (US-MB), which temporarily increases skin permeability by rapidly disrupting and reforming cell-cell junctions, has been used to enhance transdermal drug delivery. Notably, the cycle of matriptase zymogen activation induced by US-MB treatment has been observed to correspond with the mechanical disruption and subsequent restoration of cell-cell adhesion in HaCaT human keratinocytes, as well as desmosomes in human skin [75]. The importance of cell-cell adhesion in matriptase activation is further highlighted by studies using 184 A1N4 immortalized human mammary epithelial cells, where cycles of matriptase autoactivation align with the assembly of adherens junctions and cortical actin structures induced by sphingosine 1-phosphate (S1P) [48,76]. These cells rapidly deplete S1P, a bioactive blood-borne lysophospholipid, from culture medium supplemented with 0.5% fetal bovine serum. Consequently, S1P-dependent events cycle with the replacement of the medium. Upon S1P depletion, cells lose cortical actin and adherens junctions, and matriptase remains in its zymogen form. However, when fresh medium or S1P is added, matriptase rapidly translocates to newly formed cell-cell junctions, where its autoactivation is triggered.

S1P plays a critical role in various physiological processes, including cell extrusion. Its production, such as by apoptotic cells, has been proposed as the rate-limiting step in facilitating the extrusion of dying cells [77]. S1P is believed to activate S1P receptor 2 (S1PR2), initiating Rho-dependent actin and myosin contraction, which helps expel apoptotic cells from the epithelial sheet. Although the precise identity of the S1P receptor(s) involved and downstream G protein(s) remains unclear, evidence suggests that matriptase zymogen activation, along with the assembly of adherens junctions and cortical actin, can be triggered by S1P at concentrations as low as 26 nM (10 ng/mL) or culture medium, supplemented with 0.5% FBS with an estimated 2.5 nM of serum-derived S1P. At this low concentration, which is consistent with the activation range of S1P receptors, it is likely that signaling via S1P receptors and associated G proteins contributes to S1P-induced matriptase autoactivation. This implies that S1P may simultaneously promote cell extrusion and matriptase activation through shared or parallel signaling pathways. Importantly, the dynamic modulation of cell-cell adhesion, following activation of the matriptase-EpCAM/Trop-2 axes, may provide the necessary mechanism for both disconnecting dying cells from their neighbors and reestablishing new intercellular junctions among the remaining surrounding cells. These tightly coordinated processes are essential to ensure that no intercellular gaps are left behind after cell extrusion. This is an appealing hypothesis. However, despite the important findings reported by Gu et al. in 2011 identifying a critical role for S1P in cell extrusion, several aspects of the study raise important questions [77].

It appears that the S1P produced by apoptotic cells is contained within vesicle-like structures of varying sizes. Notably, this vesicle-associated S1P appears to be resistant to extraction by 0.5% Triton X-100, a detergent commonly used for cell permeabilization in immunocytochemistry [77]. This suggests that the newly generated S1P is compartmentalized within detergent-resistant domains. Moreover, the detection of S1P in this work required the use of a high concentration (50 µg/mL) of anti-S1P mAb, implying that the lipid can only be detected when extremely high levels of antibody are used. The generation of S1P in apoptotic cells is driven by the action of sphingosine kinases (SphKs), particularly SphK1. Under normal conditions, SphK1 is primarily located in the cytosol, but upon activation, translocates to the inner leaflet of the plasma membrane, where it phosphorylates sphingosine to produce S1P [78,79]. The punctate staining pattern of S1P observed in apoptotic cells supports the idea that newly synthesized S1P is incorporated into unidentified vesicular structures. It remains unclear whether healthy cells sequester S1P into highly detergent-resistant vesicles in a similar fashion, or if this is a unique feature of apoptotic cells. Alternatively, the need for such high antibody concentrations for immunocytochemistry and the detergent-resistant detection of a lysophospholipid raise the possibility that the observed S1P staining pattern in apoptotic cells could be artifactual. Furthermore, in the Gu et al. study [77], Madin-Darby Canine Kidney (MDCK) cells, were cultured in medium supplemented with 5% fetal bovine serum (FBS), which means that the culture medium likely contained at least 25 nM of serum-derived S1P, which could result in the sustained engagement of S1P receptors and potentially cause receptor desensitization through internalization. This background level of S1P may to a great extent have occupied the anti-S1P mAb used in the study at a concentration of 10 µg/mL (approximately 66.7 nM), which was intended to block apoptotic cell-induced extrusion, an important piece of evidence supporting the role of S1P in the extrusion process. However, the potential for interference caused by serum-derived S1P with the effects of apoptotic cell-derived S1P on cell extrusion was not addressed in the study.

In summary, a matriptase-driven mechanism has been identified and characterized for the rapid disruption and restoration of cell-cell adhesion involving rapid cleavage, internalization and de novo protein synthesis of EpCAM and Trop-2. Loss of matriptase disrupts the cycle, causing porous epithelial layers, defective cell extrusion, delayed division, and impaired migration. Environmental factors like pH, redox state, and chloride concentration influence matriptase activation, linking external cues to localized adhesion changes. The short active lifespan of matriptase, due to the near-instantaneous inhibition by HAI-1, ensures spatially and temporally limited CAM cleavage. This protease-regulated, non-junctional CAM remodeling complements mechanically regulated junctional systems like E-cadherin, enabling epithelial cells to swiftly adjust adhesion during tissue dynamics.

## Supplementary Material

Supplemental Materials.docx

## Data Availability

The datasets analyzed during the current study are available in the Cancer Cell Line Encyclopedia (CCLE) (xena.ucsc.edu/) and the Human Protein Atlas (https://www.proteinatlas.org/) repository. All data generated or analyzed during this study are included in this published article and its supplementary information files.
