# Supplementary material for: Dynamic modulation of intercellular adhesion mediated by matriptase-EPCAM/Trop-2 axes is critical for cell extrusion, division, and collective migration
Source: Cell Adh Migr. 2026 May 5;20(1):2663684. doi: 10.1080/19336918.2026.2663684 (PMC13154952; doi:10.1080/19336918.2026.2663684)
Supplement: Supplemental Materials.docx [file KCAM_A_2663684_SM5597.docx]

**Supplementary Materials**

*An alternative, matriptase-independent proteolytic event generates a novel Trop-2 fragment enriched in HaCaT human keratinocytes*

The forms of Trop-2 present in HaCaT keratinocytes were examined by immunoblotting under conditions that allow detection of small Trop-2 fragments and analysis of their potential interactions. Under nonreducing/nonboiled (NRNB) conditions, two Trop-2 species were detected: a 55-kDa protein (Fig. S1A, lane 1, band a) and a fragment migrating below the 15-kDa marker (Fig. S1A, lane 1, band g). The 55-kDa form retained its size under nonreducing/boiled (NRB, lane 2, band b) and reducing/boiled (RB, lane 3, band c) conditions, indicating that this species represents the mature single-chain Trop-2. The small fragment also remained unchanged under all three conditions, suggesting it does not form stable or disulfide-linked complexes with other Trop-2 species or proteins. A 35-kDa Trop-2 species, though detected at low abundance, was released by either boiling or reducing treatment (Fig. S1A, bands d and e). This fragment appeared as a broader smear than the 35-kDa Trop-2 heavy chain produced by matriptase-mediated cleavage of Trop-2 (Fig. S1B, band e vs. band f). Importantly, activation of matriptase and its cleavage of the Trop-2 N-terminus had no effect on the small Trop-2 fragment (Fig. S1B). Moreover, while the abundance of the small fragment varied across four matriptase knockout (KO) variants (Fig. S1C), no direct correlation with matriptase status was evident. In contrast to HaCaT cells, the small Trop-2 fragment was barely detectable in other cell types examined (data not shown). These findings indicate that Trop-2 undergoes an additional, matriptase-independent proteolytic cleavage, giving rise to a distinct fragment enriched in HaCaT keratinocytes.

*
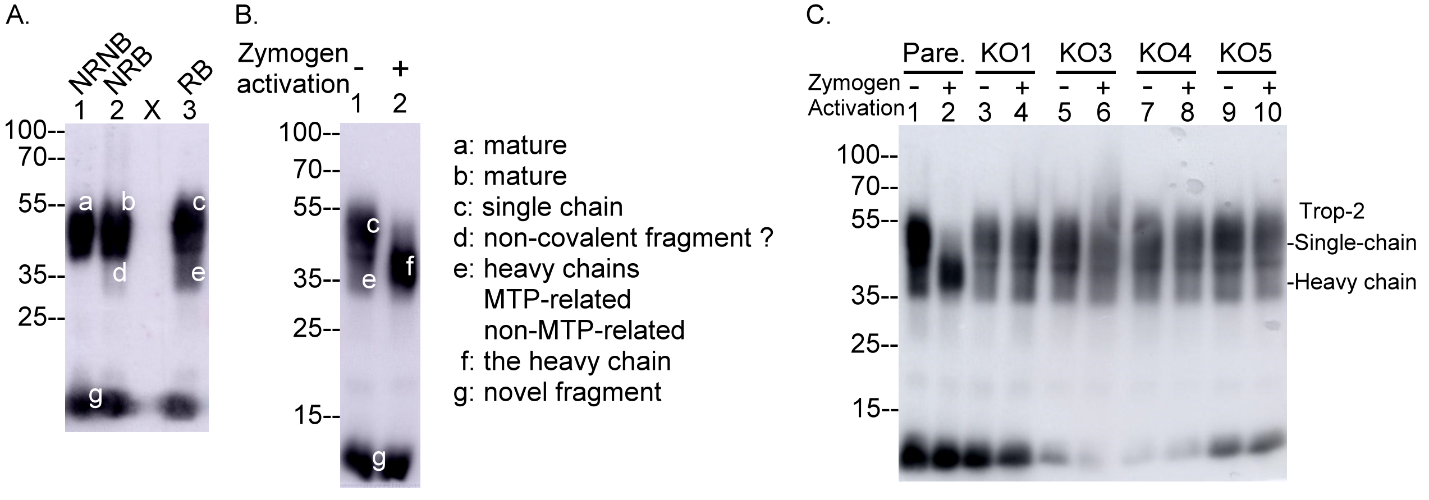
*

**Figure S1. Trop-2 undergoes an alternative, matriptase-independent proteolytic processing event that generates a novel fragment in HaCaT keratinocytes**
(A) Lysates from HaCaT keratinocytes were analyzed by immunoblotting under three conditions: non-reducing/non-boiled (NRNB), non-reducing/boiled (NRB), and reducing/boiled (RB), to assess the different Trop-2 species. (B, C) HaCaT keratinocytes and matriptase knockout (KO) variants, as indicated, were transiently treated with pH 6.0 buffer to induce matriptase zymogen activation (+) or with PBS as a control (–). Equal amounts of lysate proteins were subjected to immunoblotting for Trop-2, with the detected species labeled accordingly.

*The small Trop-2 fragment appears to localize intracellularly*Immunofluorescent staining of Trop-2 in HaCaT human keratinocytes revealed two distinct subcellular patterns: (1) localization at cell-cell interfaces, observed either as strong, sharp signals or as a more diffuse distribution, and (2) punctate staining near the perinuclear region in a subset of cells (Fig. S2A). The former reflects the expected cell-surface distribution of a cell adhesion molecule (CAM) and is thought to represent single-chain Trop-2. The latter, when considered alongside the abundant presence of the small Trop-2 fragment, suggests that the perinuclear puncta correspond to this fragment. Such punctate staining was also detected in matriptase knockout HaCaT variants (Fig. S2E), consistent with the presence of the Trop-2 fragment (Fig. S1C, lane 3) and supporting the conclusion that matriptase is not required for fragment generation. Thus, the Trop-2 fragment may arise through a combination of proteolytic cleavage and internalization by a matriptase-independent pathway, or alternatively, through intracellular cleavage.

Importantly, the perinuclear punctate staining attributed to the Trop-2 fragment complicates efforts to determine whether two-chain Trop-2 undergoes internalization upon matriptase zymogen activation. To address this, the fates of two-chain and single-chain Trop-2 were examined after transient exposure of cells to pH 6.0 buffer, followed by recovery in culture medium for 0, 15, or 120 minutes. In parental HaCaT cells, two-chain Trop-2 became more diffuse along the cell periphery immediately following matriptase activation (Fig. S2B) and then rapidly re-accumulated at intercellular junctions within 15 minutes of recovery (Fig. S2C), indicating that two-chain Trop-2 can still mediate homotypic interactions. Similarly, single-chain Trop-2 in matriptase KO1 cells accumulated at intercellular contacts, most prominently at 120 minutes of recovery (Fig. S2F–H). By contrast, only a limited fraction of two-chain Trop-2 localized at cell-cell interfaces by 120 minutes (Fig. S2D), whereas most single-chain Trop-2 did so in matriptase KO1 cells (Fig. S2H). Since punctate staining was observed in nearly all cells at 120 minutes, compared to a smaller subset at 15 minutes (Fig. S2D vs. S2C), this reduction in
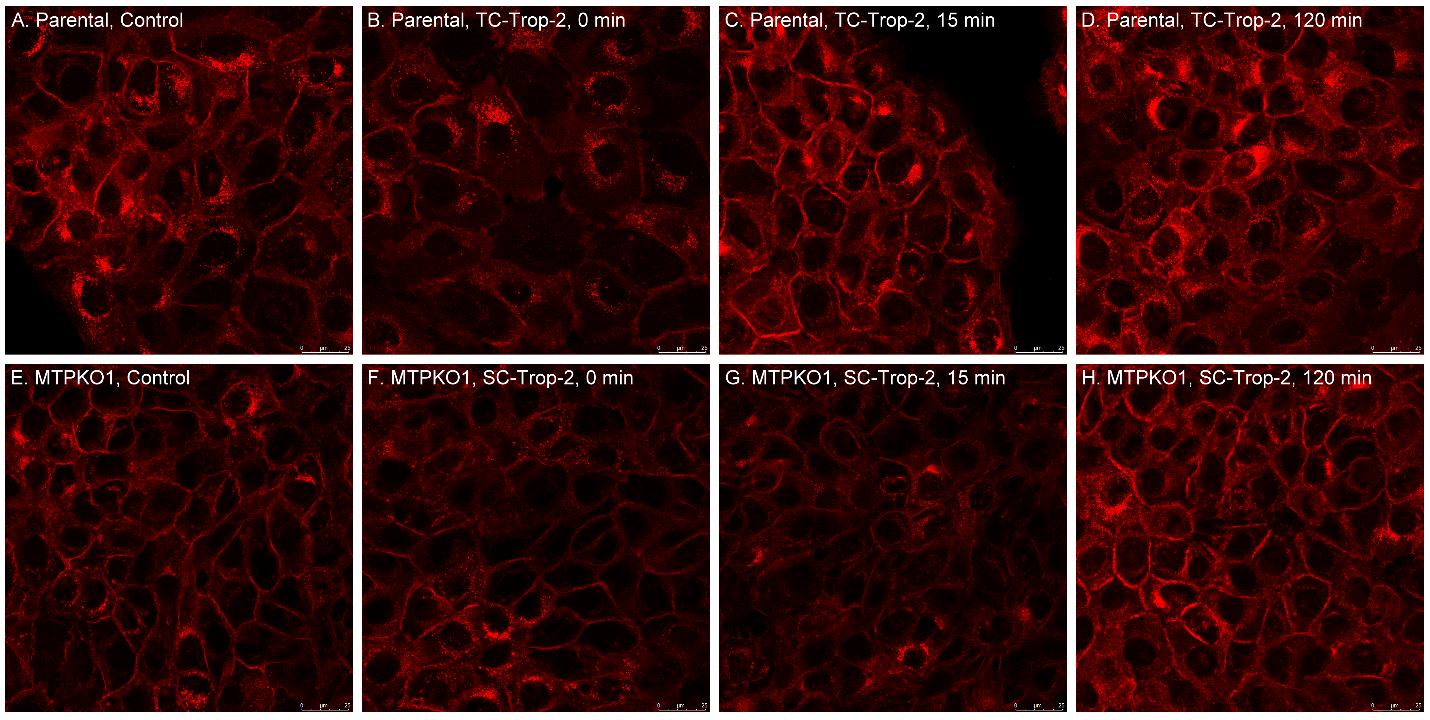
intercellular accumulation of two-chain Trop-2 is consistent with its internalization.

**Figure S2: Two-chain Trop-2 retains the ability to mediate homotypic interaction and is rapidly internalized**

Parental HaCaT cells (A-D) and a matriptase KO variant (MTPKO1, E-H) were transiently exposed to a pH 6.0 buffer, then returned to regular culture medium and incubated for the indicated time points. The cells were analyzed by fluorescent confocal microscopy to detect Trop-2 using an Trop-2 mAb and F-actin using fluorescent dye-conjugated phalloidin (data not shown). The scale bar sizes are shown.
